# Hydrogenation-controlled phase transition on two-dimensional transition metal dichalcogenides and their unique physical and catalytic properties

**DOI:** 10.1038/srep34186

**Published:** 2016-09-30

**Authors:** Yuanju Qu, Hui Pan, Chi Tat Kwok

**Affiliations:** 1 Institute of Applied Physics and Materials Engineering, Faculty of Science and Technology, University of Macau, Macao SAR, P. R. China; 2 Department of Electromechanical Engineering, Faculty of Science and Technology, University of Macau, Macao SAR, P. R. China

## Abstract

Two-dimensional (2D) transition metal dichalcogenides (TMDs) have been widely used from nanodevices to energy harvesting/storage because of their tunable physical and chemical properties. In this work, we systematically investigate the effects of hydrogenation on the structural, electronic, magnetic, and catalytic properties of 33 TMDs based on first-principles calculations. We find that the stable phases of TMD monolayers can transit from 1T to 2H phase or vice versa upon the hydrogenation. We show that the hydrogenation can switch their magnetic and electronic states accompanying with the phase transition. The hydrogenation can tune the magnetic states of TMDs among non-, ferro, para-, and antiferro-magnetism and their electronic states among semiconductor, metal, and half-metal. We further show that, out of 33 TMD monolayers, 2H-TiS_2_ has impressive catalytic ability comparable to Pt in hydrogen evolution reaction in a wide range of hydrogen coverages. Our findings would shed the light on the multi-functional applications of TMDs.

Extensive attention has been drawn to two-dimensional (2D) transition metal dichacogenides (TMDs) because of their unique chemical, mechanical, electronic and magnetic properties, multi-functional applications in various fields of science and technology from spintronics, optoelectronics, sensors, catalysts to energy harvesting and storage^1^,^2^,^3^,^4^,^5^,^6^,^7^,^8^,^9^,^10^,^11^,^12^,^13^,^14^, and easier fabrication^2^,^15^,^16^,^17^,^18^. Depending on the point-group symmetries (D_6h_ and D_3d_), these 2D monolayers with the formula of MX_2_ can have 1T (Fig. 1a) or 2H phase (Fig. 1d)^2^, where M is the transition metal element and X is a chalcogen element (S, Se, and Te). These 2D TMDs show rich physical and chemical properties and can be metallic, half-metallic, semiconducting, magnetic, and catalytic, which can be tuned by phase transition, composition engineering, surface functionalization, and external fields (strain and electrical field)^2^,^9^,^19^,^20^,^21^,^22^,^23^,^24^,^25^,^26^,^27^,^28^,^29^,^30^,^31^,^32^. For example, 2H MoS_2_ and WS_2_ monolayers are semiconductor, while their 1T phases are metallic^32^,^33^,^34^. Hydrogenated MoS_2_ monolayer can be non-magnetic and ferromagnetic tuned by tensile strain^23^. Magnetic evolution from non-magnetism, to anti-ferromagnetism, via paramagnetism, then to ferromagnetism accompanying with electronic switching from semiconductor to metal, then to half-metal was achieved on VX_2_ monolayers by hydrogenation and tensile strain^11^,^24^. Vanadium disulfide monolayer showed better catalytic performance than its selenides and tellurides counterparts^25^ and tensile strain can enhance the ability dramatically^26^. The catalytic performance of MX_2_ monolayers can be strongly improved by phase transition^30^,^31^,^32^,^33^,^34^,^35^,^36^,^37^,^38^. For example, the 1T phase MoS_2_ and WS_2_ nanosheet was proven to be more catalytically active in facilitating hydrogen production in electrolysis of water than their 2H counterpart, although 2H phase is more stable than its 1T phase^30^. Theoretically, it was reported that hydrogen-functionalization can trigger the 2H to 1T phase transition of MoS_2_^28^ and the catalytic activity of 1T-MoS_2_ mainly arises from its affinity for binding H at the surface S sites^28^,^39^,^40^,^41^. Recently, 1T phase domains were formed in 2H-MX_2_ monolayer by creating X vacancies and these 2H-1T mixed monolayer showed ferromagnetism^31^. Although oxidization is a simple way to create the vacancy for the phase transition, it may also result in a lot of defects in the monolayers. In this work, we present a general method – hydrogenation – to realize the phase transition and tune the physical and chemical properties of 33 different MX_2_ monolayers. We find that 1T and 2H phases can transform to each other upon hydrogenation, depending on the transition metal elements in MX_2_ monolayers. Accompanying with the phase transition, their electronic properties switch among semiconducting, metallic, and half metallic, and magnetic ground states among nonmagnetic, ferromagnetic, paramagnetic and anti-ferromagnetic states. We further predict that TiS_2_ monolayer in 2H phase shows effective catalytic performance for hydrogen evolution reaction in a wide range of hydrogen coverages with neutral thermal Gibbs free energies.

## Results and Discussion

### Structural Properties

**Figure 1:**
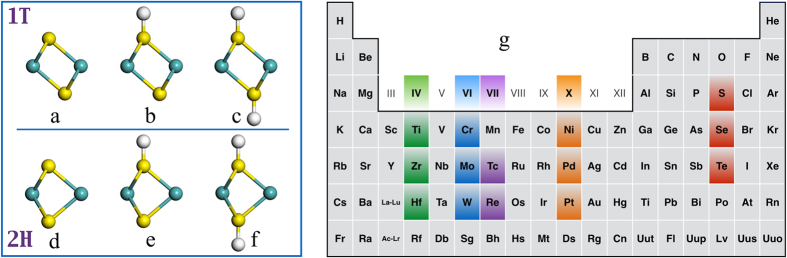
The representative unit cells of MX_2_ monolayers in 1T phase: (**a**) MX_2_-0HC, (**b**) MX_2_-1HC, (**c**) MX_2_-2HC. The representative structures of MX_2_ monolayers unit cell in 2H phase: (**d**) MX_2_-0HC, (**e**) MX_2_-1HC, (**f**) MX_2_-2HC. (**g**) the periodic table with highlighted transition metal atoms (M) and chalcogen atoms (X) considered in our calculations.

In our calculations, we focus on 2D transitional metal dichalcogenides (MX_2_) with M from group IV (Ti, Zr, and Hf), group VI (Cr, Mo, and W), group VII (Tc and Re), and group VIII (Ni, Pd, and Pt), as shown in Fig. 1g. The hydrogenation of these TMDs is realized by putting hydrogen atoms directly on the tops of X atoms^41^,^42^,^43^,^44^. The MX_2_ monolayers with and without hydrogenation is referred as MX_2_-nHC, where n equals to 0 (no hydrogenation; Fig. 1a for 1T phase and Fig. 1d for 2H phase, respectively), 1 (one surface fully covered by hydrogen atoms; Fig. 1b for 1T phase and Fig. 1e for 2H phase, respectively), and 2 (two surfaces fully covered by hydrogen atoms; Fig. 1c,f for 1T and 2H phases, respectively). In order to identify the phase transition of 2D TMDs, all the MX_2_ unit cells with and without hydrogenation in both 1T and 2H phases (Fig. 1) are firstly optimized to obtain the lattice parameters and formation energies. The phase transition can be identified by the energy differences (ΔE_1T−2H_) between 1T and 2H phases of MX_2_ as calculated from the following equation:


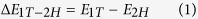


where E_1T_ and E_2H_ are the total energies of a MX_2_-nHC in 1T and 2H phases, respectively. If ΔE_1T−2H_ is negative, the 1T phase is more stable, otherwise 2H phase is more stable.

**Figure 2:**
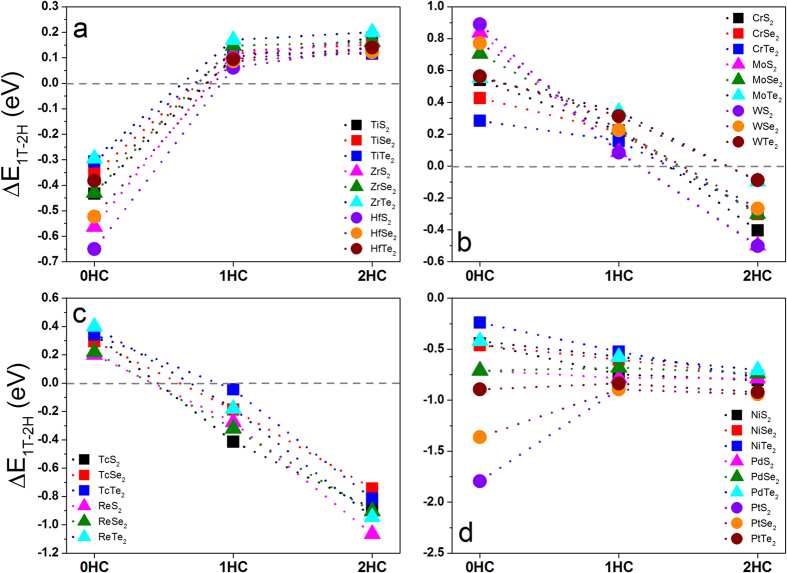
Calculated total energy differences between 1T and 2H phase of MX_2_ monolayers with and without hydrogenation for metal elements from: (**a**) group IV: M = Ti, Zr & Hf, (**b**) group VI: M = Cr, Mo & W, (**c**) group VII: M = Tc & Re, (**d**) group X: M = Ni, Pd & Pt.

The negative ΔE_1T−2H_ shows that pure MX_2_ monolayers with metal elements from group IV (M = Ti, Zr, and Hf) are stable in 1T phase (Fig. 2a). When their surfaces are hydrogenated, the 2H phases become stable, as indicated by the positive ΔE_1T−2H_. We also note that 1T transition metal disulfides (MS_2_) are more stable than their selenides (MSe_2_) and tellurides (MTe_2_) with the same phase due to their larger negative energy differences. Differently, the MX_2_ monolayers with the metal elements from group VI (M = Cr, Mo, and W) experience a phase transition of 2H → 2H → 1T as the hydrogenation progresses from 0HC, 1HC, to 2HC (Fig. 2b). Our result on MoS_2_ is consistent with literatures^35^,^36^,^37^. From literatures^35^,^36^,^37^, we also see that pure 2H-MoS_2_ is more stable than pure 1T-MoS_2_. As hydrogen coverage increasing, 1T-MoS_2_ becomes stable^28^. If the metal element is from group VII, the MX_2_ (M = Tc and Re) monolayers go through a phase transition of 2H → 1T → 1T as the hydrogenation increases from 0HC, 1HC, to 2HC (Fig. 2c). Totally different from the above three groups, all of the MX_2_ monolayers with metal elements from group X (M = Ni, Pd, and Pt) are stable in 1T phase regardless of hydrogenation due to large negative energy differences. Our calculations show that pure MX_2_ monolayers are stable in either 1T or 2H phase depending on the metal elements in their composition, and hydrogenation can trigger the phase transition from one to another effectively, except the MX_2_ monolayers with M from group X. The MX_2_ monolayers, where M atoms are in the same group, follow the same trend of phase transition under hydrogenation.

In the following discussion, the pure and hydrogenated MX_2_ monolayers with the stable phases are investigated (Table 1 and Fig. 2). The optimized geometries show that the hydrogenation results more or less in the changes of their lattice constants. For MX_2_ with M from group IV, their lattice constants (a) and the X-M bond length remain unchanged or slightly decrease under hydrogenation. For example, the lattice constant (a) are 3.47 Å for both TiS_2_-1HC and TiS_2_-2HC, slightly larger than that of TiS_2_-0HC (3.42 Å). The lattice constant of pure TiS_2_ monolayer is consistent with literature^45^. Normally, the thickness (the vertical distance between two chalcogen atoms) increases upon hydrogenation in all considered MX_2_ systems. For MX_2_ monolayers with M from group VI, VII, and X, both the lattice constant (a) and X-M bond length increase as hydrogenation increases from 0HC, 1HC, to 2HC. For example, the lattice constants are extended by 8–9%, 1–9%, and 0–10% for CrX_2_, MoX_2_, and WX_2_, respectively, under hydrogenation.

### Magnetic Properties

Our calculations show that the hydrogenation results in the phase transition of TMD monolayers, which may also tune other physical properties. In this section, we focus on the effect of hydrogenation on the magnetic properties of the TMD monolayers. To find the magnetic ground state, a supercell with 2 × 2 × 1 unit cells for each MX_2_ system is constructed (referred as 221 supercell in Supporting Data S1). As an indication of stable ground state, the exchange energy (E_ex_) is calculated as below:


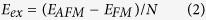


where E_AFM_ and E_FM_ are the total energies of a MX_2_ monolayer at antiferromagnetic and ferromagnetic states, respectively, and N is the number of unit cells adopted in calculation (N = 4 in a 221 supercell). The MX_2_ monolayer is ferromagnetic (FM) when E_ex_ is positive, while antiferromagnetic (AFM) when E_ex_ is negative. We consider the systems as non-magnetism (NM) when the absolute values of E_ex_, E_AFM_ − E_NM_, and E_FM_ − E_NM_ are less than 10 meV per unit cell because of possible calculation error.

**Figure 3:**
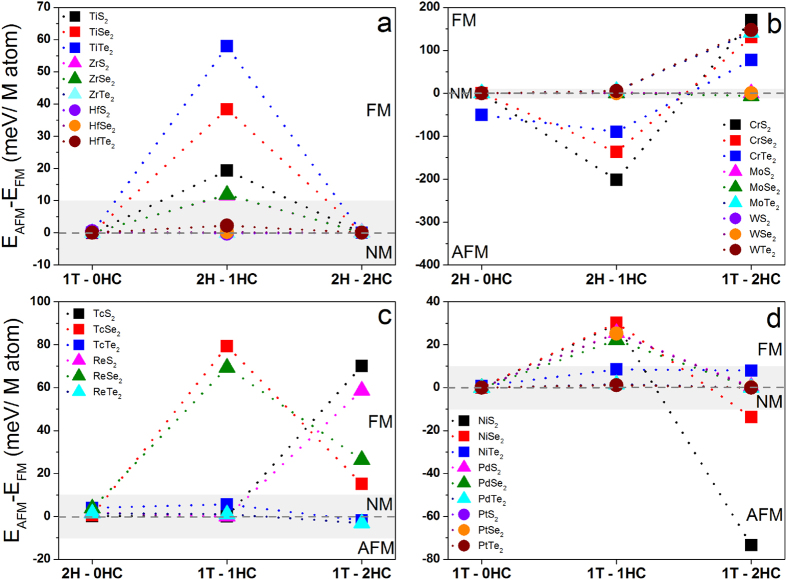
Calculated exchange energies between antiferromagnetic and ferromagnetic states of MX_2_ monolayers with and without hydrogenation for metal elements from: (**a**) group IV: M = Ti, Zr & Hf, (**b**) group VI: M = Cr, Mo & W, (**c**) group VII: M = Tc & Re, (**d**) group X: M = Ni, Pd & Pt. The grey region indicates the non-magnetic states.

The calculated exchange energies show that nonmagnetic TiX_2_, ZrS_2_ and ZrSe_2_ monolayers switch to ferromagnetic, then back to nonmagnetic as the hydrogenation progresses from 0HC, 1HC, to 2HC (Fig. 3a). Whereas, ZrTe_2_ and HfX_2_ monolayers are nonmagnetic regardless of the hydrogenation. The Curie temperatures (T_C_) of ferromagnetic systems can be estimated from K_B_T_C_ = (2/3)E_ex_ based on the mean field theory and Heisenberg model^46^, which are 448, 297, 149, 90, and 91 K for TiTe_2_-1HC, TiSe_2_-1HC, TiS_2_-1HC, ZrS_2_-1HC and ZrSe_2_-1HC, respectively (Table 2).

For MX_2_ monolayers with M from group VI, the magnetic switching under hydrogenation is slightly complicated. CrS_2_ and CrSe_2_ switch following NM → AFM → FM with the hydrogenation increasing (Fig. 3b). CrTe_2_-0HC and CrTe_2_-1HC are antiferromagnetic, but CrTe_2_-2HC is ferromagnetic. MX_2_ (M = Mo and W) monolayers with and without hydrogenation are nonmagnetic, except MoTe_2_-2HC and WTe_2_-2HC are ferromagnetic because their exchange energies are positive (Fig. 3b) and 1T-MoSe_2_-2HC is paramagnetic because the energies at its magnetic states are lower than that at non-magnetic state (Supporting data, Table S1). Importantly, the Curie temperatures of CeS_2_-2HC, CrSe_2_-2HC, MoTe_2_-2HC, and WTe_2_-2HC are above 1000 K (Table 3).

For MX_2_ monolayers with M from VII, we see that MTe_2_ (M = Tc and Re) are nonmagnetic regardless of hydrogenation (Fig. 3c), except 1T-TcTe_2_-2HC is paramagnetic because its magnetic states are more stable than its non-magnetic state (Supporting Data, Table S1). For systematic study, Tc is considered although it is radioactive. MSe_2_ switches from non-magnetism to ferromagnetism after hydrogenation. MS_2_-0HC and MS_2_-1HC are non-magnetic, while MS_2_-2HC monolayers are ferromagnetic (Fig. 3c and Table 4). The Curie temperatures range from 117 to 613 K for ferromagnetic systems in this group.

For MX_2_ with M from group X, NiS_2_ and NiSe_2_ show the same magnetic evolution as NM → FM → AFM with the hydrogenation increasing (0HC → 1HC → 2HC), whereas NiTe_2_ is nonmagnetic regardless of hydrogenation (Fig. 3d and Table 5). The ground states of MS_2_ and MSe_2_ (M = Pd and Pt) switch as NM → FM → NM with the hydrogenation increasing (0HC → 1HC → 2HC). However, MTe_2_ keeps NM unchanged under hydrogenation. The Curie temperatures of ferromagnetic systems in this group varies from 171 to 233 K.

Our calculations show that the hydrogenation induces not only the phase transitions of MX_2_ monolayers, but magnetic switching. Among all 33 considered systems, CrS_2_-2HC has the highest Tc (1317 K). The estimated Curie temperature needs to be confirmed experimentally. The ferromagnetic MX_2_ systems with Tc above room temperature may find applications in spintronics. To reveal the origin of the magnetic switching, their electronic structures are calculated.

### Electronic Properties

**Figure 4:**
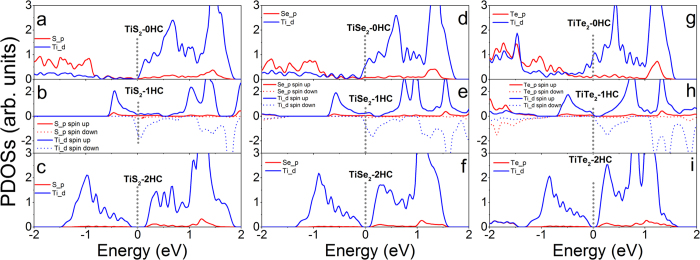
Calculated partial density of states of (**a**) TiS_2_-0HC, (**b**) TiS_2_-1HC, (**c**) TiS_2_- 2HC, (**d**) TiSe_2_-0HC, (**e**) TiSe_2_-1HC, (**f**) TiSe_2_-2HC, **(g**) TiTe_2_-0HC, (**h**) TiTe_2_-1HC and (**i**) TiTe_2_-2HC monolayers. The Fermi level is at 0 eV and indicated with gray dotted line.

To understand the magnetic evolution of MX_2_ monolayers under hydrogenation, the electronic structures and magnetic moments of MX_2_ monolayers with and without hydrogenation are calculated (Figs 4 and 7, S2~S12, and Tables 2~5). The calculated partial density of states (PDOSs) of TiX_2_-nHC show that nonmagnetic TiX_2_-0HC and TiX_2_-2HC monolayers are either metallic or semiconducting (Figs 4a,c and S2a,c), while ferromagnetic TiS_2_-1HC and TiSe_2_-1HC monolayers are n-type semiconductors and TiTe_2_-1HC is narrow band semiconductor (Figs 4b,e,h and S2b,e,h). The d electrons of Ti atoms near the Fermi levels are spin-polarized (Fig. 4b,e,h), leading to the magnetic moments of 0.46, 0.62, and 0.71 μ_B_/Ti in TiX_2_-1HC (X = S, Se, and Te), respectively (Table 2). The p electrons of X atoms in TiX_2_-1HC are also weakly spin-polarized (Fig. 4b,e,h), leading to smaller magnetic moments (Table 2), which are anti-parallel to the moments of Ti atoms. Therefore, the ferromagnetism of TiX_2_-1HC may attribute to the double-exchange^11^,^14^,^47^,^48^,^49^,^50^. Pure ZrS_2_ and ZrSe_2_ monolayers are semiconductors and their band gaps are reduced by two-surface hydrogenation (S3). The ferromagnetic ZrS_2_-1HC and ZrSe_2_-1HC are half-metal (S3b,e). Nonmagnetic ZrTe_2_ switches from metal, n-type semiconductor, to intrinsic semiconductor as hydrogenation increases from 0HC, 1HC, and 2HC (S3g–i). Pure nonmagnetic HfS_2_ and HfSe_2_ monolayers are semiconductors, and switch to metal upon hydrogenation (S4b,c,e,f), except that HfS_2_-2HC is narrow-band semiconductor (S4c). Nonmagnetic HfTe_2_ monolayer keeps metallic regardless of hydrogenation (S4g,h,i).

**Figure 5:**
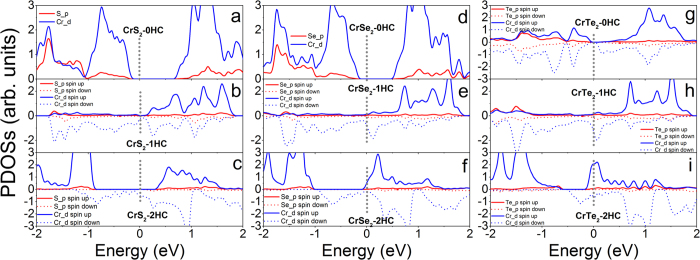
Calculated partial density of states of (**a**) CrS_2_-0HC, (**b**) CrS_2_-1HC, (**c**) CrS_2_- 2HC, (**d**) CrSe_2_-0HC, (**e**) CrSe_2_-1HC, (**f**) CrSe_2_-2HC, (**g**) CrTe_2_-0HC, (**h**) CrTe_2_-1HC and (**i**) CrTe_2_-2HC monolayers. The Fermi level is at 0 eV and indicated with gray dotted line.

For MX_2_ with M from group VI, accompanying with the magnetic switching of NM → AFM → FM, the electronic properties of CrX_2_ monolayers switch from semiconductor, metal, to half-metal as hydrogenation increases (0HC → 1HC → 2HC) (Figs 5 and S5). Nonmagnetic MoX_2_ (X = S and Se) systems switch from intrinsic semiconductor, n-type semiconductor, to metal as hydrogenation increases (S6a–f). Semiconducting MoTe_2_ monolayer transfers to metal upon 1HC, and to half-metal upon 2HC (S6g–i). WX_2_ monolayers show the same electronic switching as MoX_2_ upon hydrogenation (S7).

**Figure 6:**
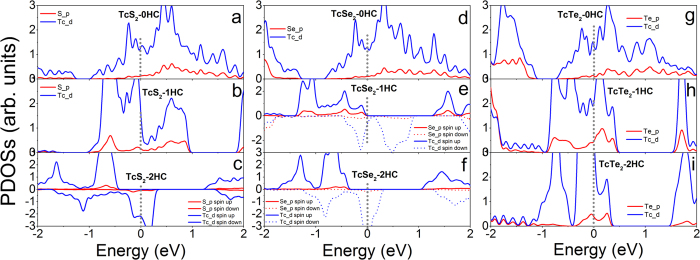
Calculated partial density of states of (**a**) TcS_2_-0HC, (**b**) TcS_2_-1HC, (**c**) TcS_2_- 2HC, (**d**) TcSe_2_-0HC, (**e**) TcSe_2_-1HC, (**f**) TcSe_2_-2HC, (**g**) TcTe_2_-0HC, (**h**) TcTe_2_-1HC and (**i**) TcTe_2_-2HC monolayers. The Fermi level is at 0 eV and indicated with gray dotted line.

For MX_2_ with M from group VII, nonmagnetic TcS_2_-0HC and TcS_2_-1HC are metallic, and ferromagnetic TcS_2_-2HC is half-metallic (Figs 6a–c and S8). Nonmagnetic TcSe_2_-0HC is metal (Fig. 6d) and ferromagnetic TcSe_2_-1HC and TcSe_2_-2HC are half-metals, respectively (Fig. 6e,f). Nonmagnetic TcTe_2_ monolayers with and without hydrogenation are metallic (Figs 6g–i and S8). The nonmagnetic ReX_2_ monolayers with and without hydrogenation are metallic (S9a,b,d,g–i), while ferromagnetic counterparts are half-metallic (S9c,e,f).

**Figure 7:**
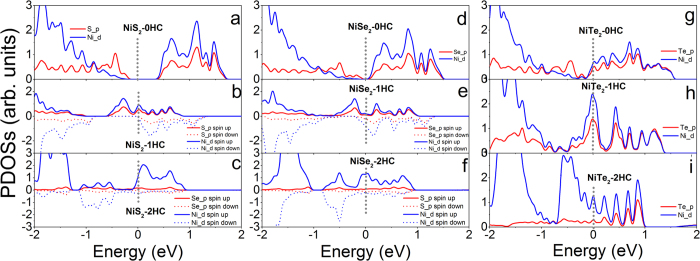
Calculated partial density of states of (**a**) NiS_2_-0HC, (**b**) NiS_2_-1HC, (**c**) NiS_2_- 2HC, (**d**) NiSe_2_-0HC, (**e**) NiSe_2_-1HC, (**f**) NiSe_2_-2HC, (**g**) NiTe_2_-0HC, (**h**) NiTe_2_-1HC and (**i**) NiTe_2_-2HC monolayers. The Fermi level is at 0 eV and indicated with gray dotted line.

For MX_2_ with M from group X, semiconducting NiX_2_ (X = S and Se) monolayers switch to half-metal upon 1HC (Figs 7b,e and S10b,e), and metallic upon 2HC (Figs 7c,f and S10c,f). Nonmagnetic NiTe_2_ systems keep metallic regardless of hydrogenation (Figs 7g–i and S10 g–i). Nonmagnetic MX_2_-0HC and MX_2_-2HC monolayers (M = Pd and Pt, X = S and Se) are semiconducting and metallic, respectively (S12a,c,d,f, and S13a,c,d,f), and MX_2_-1HC monolayers are ferromagnetic n-type semiconductors (S11b,e and S12b,e).

The calculated electronic properties clearly show that metallic or semiconducting systems are nonmagnetic/antiferromagnetic, and half metallic or doped semiconducting systems are ferromagnetic. The ferromagnetism is contributed to the carrier-mediated double exchange^11^,^24^,^47^,^48^,^49^,^50^.

### Catalytic Ability for Hydrogen Evolution

MX_2_ monolayers have been widely investigated as electrocatalysts for hydrogen evolution reaction (HER)^25^,^26^,^32^,^33^,^34^,^42^,^43^,^44^,^51^,^52^,^53^,^54^,^55^,^56^,^57^,^58^,^59^,^60^. In this section, we investigate the catalytic activities of the considered 33 MX_2_ systems. To characterize their catalytic performances, Gibbs free energies (∆G_H_) are calculated based on published methods^43^,^53^,^54^,^60^. A catalyst with optimal performance needs to have near-zero ∆G_H_. Two HER processes, individual and collective processes, are considered. I-ΔG_H_ and A-ΔG_H_ are referred as the Gibbs free energies calculated from individual and collective processes, respectively^26^,^53^. To investigate the catalytic activities of the 33 MX_2_ monolayers in HER, a supercell with 3 × 3 × 1 unit cells for each MX_2_-1HC monolayer, referred as 331 supercell, is constructed (S13). Hydrogen atoms are taken away one by one from 331 supercell to calculate the Gibbs free energies, where the partial hydrogen coverages is referred as 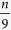 (n = 1–9). Both 1T and 2H phases are considered because of the possible phase transition as discussed above.

**Figure 8:**
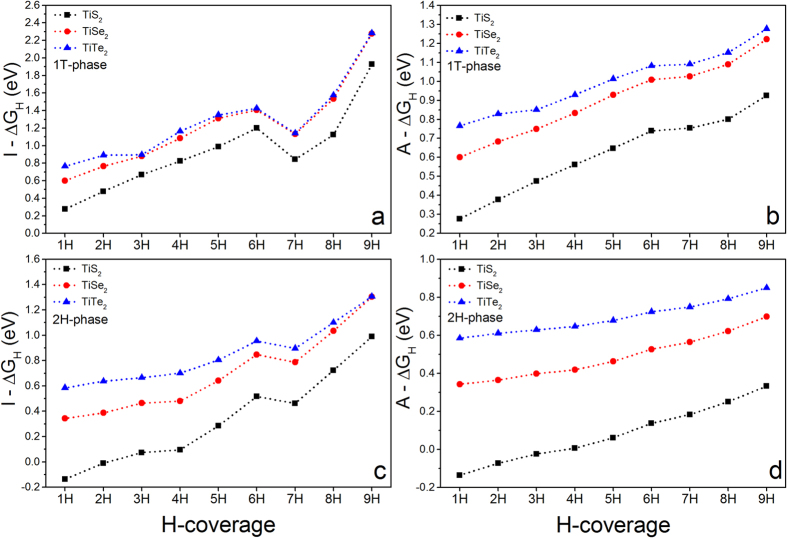
Calculated Gibbs free energies of TiS_2_ monolayer as a function of one-surface hydrogen coverages in 1T phase: (**a**) I-ΔG_H_, and (**b**) A-ΔG_H_, in 2H phase: (**c**) I-ΔG_H_, and (**d**) A-ΔG_H_.

Out of 33 MX_2_ monolayers, we find that TiS_2_ monolayer shows excellent catalytic ability at a wide range of hydrogen coverages. Our calculations show that both I-ΔG_H_ and A-ΔG_H_ of TiS_2_-1HC increase with the increment of hydrogen coverages (Fig. 8a–d), which are much closer to zero than those of TiSe_2_ and TiTe_2_ at the same hydrogen coverages, similar to VX_2_ monolayers^25^. Interestingly, we see that 2H phase shows much better catalytic performance than 1T phase because of the relatively lower overpotentials (absolute value of Gibbs free energy) at the same hydrogen coverages. As discussed above, 1T phase of pure TiX_2_ is more stable than its 2H phase, indicating that the metastable phase shows better catalytic performance, which is similar to MoS_2_ and WS_2_ monolayers^30^,^32^,^33^. At the same time, we showed above that the phase transition from 1T to 2H can be easily realized through surface hydrogenation. Therefore, 2H-TiX_2_ could be achieved and stabilized during the process of HER. Importantly, I-ΔG_H_ of 2H-TiS_2_ is close-to-zero (−0.14, −0.01, 0.07, and 0.09 eV) in a hydrogen coverage ranging from 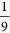 to 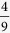 in the individual process. For collective process, A-ΔG_H_ (−0.14, −0.07, −0.02, 0.006, and 0.06 eV) is near-zero in a hydrogen density of from 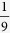 to 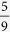. The near-zero Gibbs free energies clearly indicate that 2H-TiS_2_ monolayer show high catalytic ability within a wide range of hydrogen coverages. The catalytic activity of 2H-TiS_2_ monolayer is much better than other MX_2_ that only showed catalytic activity at certain hydrogen coverage. For example, VS_2_ monolayer was good at low-hydrogen coverages^25^. The Mo-edge of MoS_2_ was only catalytically active with an A-ΔG_H_ of 0.06 eV at a hydrogen density of 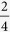
44. 1T-WS_2_ monolayer has an I-ΔG_H_ of 0.28 eV at a hydrogen density of 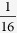
30. Therefore, TiS_2_ monolayer possesses overall excellent catalytic ability, which is comparable to Pt, due to near-zero ΔG_H_ in wide range of hydrogen densities.

## Conclusions

**Figure 9:**
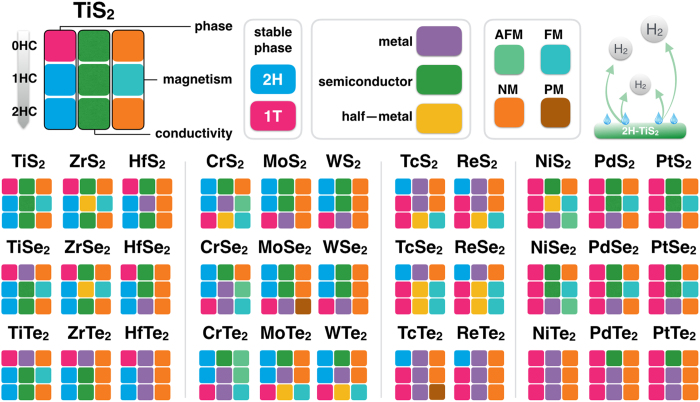
Summary of our findings on the structural, magnetic, electronic and catalytic properties of 33 TMDs monolayers with and without hydrogenation.

In summary, we present a comprehensive first-principles calculations on the physical and chemical properties of 2D TMDs with and without hydrogenation. We find that the hydrogenation plays an important role on tuning the structural, electronic, magnetic and catalytic properties of TMD monolayers (Fig. 9). We show that pure 1T-MX_2_ (M in group IV) monolayers transfer into 2H phase upon hydrogenation, their ground states can be tuned from non-magnetic to ferromagnetic accompanying with electronic switching from semiconducting to half-metallic. Phase transition between 1T and 2H phases in MX_2_ (M is from group VI and VII), and magnetic and electronic switching in MX_2_ (M is from group VI, VII, X) can be realized by the hydrogenation. We further predict that 2H-TiS_2_ monolayer, out of 33 MX_2_ monolayers, has excellent catalytic ability in a wide range of hydrogen coverage and may find applications as electrocatalyst in hydrogen evolution reaction. It is expected that MX_2_ monolayers with controllable structure, tunable electronic, magnetic and optimized catalytic properties can find applications in catalysts, spintronics, sensors and nanodevices.

## Methods

The first-principles calculations are conducted to systematically investigate the structural, electronic and magnetic properties of 2D TMDs monolayers through hydrogenation as well as their catalytic ability in hydrogen evolution reduction. Based on the density functional theory (DFT)^61^ and the Perdew-Burke-Eznerhof generalized gradient approximation (PBE-GGA)^62^, our calculations are carried out by using the Vienna ab initio simulation package (VASP)^63^, which is incorporated with projector augmented wave (PAW) scheme^64^,^65^. An energy cut-off of 500 eV is consistently used in our calculations. Large vacuum regions of 20 Å along vertical directions are used in constructing the unit cells to avoid interaction between neighboring monolayers. The integration over the first Brillouin zone is based on the Monkhorst and Pack scheme of k-point sampling^66^. The 13 × 13 × 1 grid, 5 × 5 × 1 grid, and 3 × 3 × 1 grid for k-point sampling are used for geometry relaxation of unit cells, 2 × 2 × 1 supercells, and 3 × 3 × 1 supercells, respectively. Spin-polarized calculations are also employed to study the magnetic properties. Good Convergence is obtained with these parameters and the total energy is converged to 2.0 × 10^−5 ^eV/atom.

The Gibbs free energy is an important descriptor for electrocatalyst in HER, and can be calculated as ΔG_H_ = ΔE_H_ + ΔE_ZPE_ − TΔS_H_, where ΔE_H_ is the hydrogen chemisorption energy defined as I-ΔE_H_ = E(MX_2_ + mH) − E(MX_2_ + (m–1)H) − E(H_2_)/2 for individual process and A-ΔE_H_ = [E(MX_2_ + mH) − E(MX_2_) − mE(H_2_)/2 ]/m for average process, respectively. E(H_2_), E(MX_2_) and E(MX_2_ + mH) are the calculated total energies of a hydrogen molecule, pure MX_2_ and hydrogenated MX_2_, m is the number of hydrogen atoms adsorbed on a monolayer. ΔE_ZPE_ is the difference in zero point energy between the adsorbed and the gas phase, ΔS_H_ is the difference in entropy, ΔE_ZPE_ − TΔS_H_ is about 0.24 eV^25^,^42^,^43^,^44^,^45^,^53^. Therefore, Gibbs free energy can be calculated as ΔG_H_ = ΔE_H_ + 0.24. Positive ΔG_H_ of a catalyst suggests weak adsorption of protons, leading to absorbing less protons on its surface, however, negative ΔG_H_ indicates strong binding of protons on a catalyst’s surface, resulting in difficult desorption. Electrocatalysts with neutral-thermal (close-to-zero) ΔG_H_ exhibit optimal catalytic ability in hydrogen evolution with neither stronge nor weak binding of protons in the electrolyte.

## Additional Information

**How to cite this article**: Qu, Y. *et al*. Hydrogenation-controlled phase transition on two-dimensional transition metal dichalcogenides and their unique physical and catalytic properties. *Sci. Rep.* **6**, 34186; doi: 10.1038/srep34186 (2016).

## Supplementary Material

Supplementary Information

## Figures and Tables

**Table 1:** Stable phases and lattice parameters of MX_2_ monolayers without hydrogenation (0HC), with one-surface full hydrogenation (1HC) and two-surface full hydrogenation.

Group IV	Condition	Stable Phase	a (Å)	X – M (Å)	Thickness (Å)	Group VI	Condition	Stable Phase	a (Å)	X – M (Å)	Thickness (Å)
TiS_2_	0HC	1T	3.42	2.43	2.83	CrS_2_	0HC	2H	3.04	2.29	2.94
	1HC	2H	3.47	2.45	4.18		1HC	2H	3.31	2.31	3.97
	2HC	2H	3.47	2.45	5.57		2HC	1T	3.43	2.34	5.25
TiSe_2_	0HC	1T	3.54	2.56	3.10	CrSe_2_	0HC	2H	3.21	2.43	3.15
	1HC	2H	3.64	2.58	4.50		1HC	2H	3.49	2.45	4.32
	2HC	2H	3.65	2.58	6.01		2HC	1T	3.67	2.48	5.62
TiTe_2_	0HC	1T	3.75	2.78	3.49	CrTe_2_	0HC	2H	3.47	2.64	3.42
	1HC	2H	3.93	2.79	4.97		1HC	2H	3.75	2.65	4.78
	2HC	2H	3.93	2.78	6.66		2HC	1T	3.99	2.66	6.10
ZrS_2_	0HC	1T	3.69	2.57	2.89	MoS_2_	0HC	2H	3.19	2.41	3.13
	1HC	2H	3.67	2.59	4.34		1HC	2H	3.22	2.44	4.57
	2HC	2H	3.60	2.59	5.83		2HC	1T	3.44	2.44	5.62
ZrSe_2_	0HC	1T	3.80	2.70	3.16	MoSe_2_	0HC	2H	3.32	2.54	3.34
	1HC	2H	3.82	2.71	4.66		1HC	2H	3.38	2.57	4.92
	2HC	2H	3.77	2.71	6.26		2HC	1T	3.65	2.58	5.99
ZrTe_2_	0HC	1T	3.97	2.92	3.61	MoTe_2_	0HC	2H	3.56	2.74	3.62
	1HC	2H	4.08	2.91	5.12		1HC	2H	3.90	2.74	4.86
	2HC	2H	4.04	2.90	6.87		2HC	1T	4.04	2.75	6.35
HfS_2_	0HC	1T	3.64	2.55	2.89	WS_2_	0HC	2H	3.19	2.42	3.14
	1HC	2H	3.62	2.56	4.32		1HC	2H	3.19	2.44	4.64
	2HC	2H	3.56	2.56	5.81		2HC	1T	3.40	2.44	5.68
HfSe_2_	0HC	1T	3.77	2.68	3.14	WSe_2_	0HC	2H	3.32	2.55	3.36
	1HC	2H	3.78	2.69	4.64		1HC	2H	3.34	2.57	4.99
	2HC	2H	3.70	2.68	6.30		2HC	1T	3.61	2.57	6.11
HfTe_2_	0HC	1T	3.98	2.90	3.52	WTe_2_	0HC	2H	3.56	2.74	3.63
	1HC	2H	4.04	2.89	5.11		1HC	2H	3.92	2.74	4.83
	2HC	2H	3.99	2.87	6.88		2HC	1T	4.06	2.76	6.40
											
**Group VI**						**Group X**					
TcS_2_	0HC	2H	3.28	2.38	2.89	NiS_2_	0HC	1T	3.36	2.26	2.32
	1HC	1T	3.68	2.41	3.60		1HC	1T	3.46	2.31	3.69
	2HC	1T	3.63	2.41	5.16		2HC	1T	3.63	2.37	4.96
TcSe_2_	0HC	2H	3.42	2.51	3.10	NiSe_2_	0HC	1T	3.54	2.39	2.48
	1HC	1T	3.85	2.53	3.93		1HC	1T	3.66	2.44	3.96
	2HC	1T	3.87	2.54	5.45		2HC	1T	3.81	2.49	5.37
TcTe_2_	0HC	2H	3.67	2.69	3.33	NiTe_2_	0HC	1T	3.79	2.58	2.73
	1HC	1T	4.05	2.70	4.41		1HC	1T	3.90	2.61	4.39
	2HC	1T	4.12	2.70	5.98		2HC	1T	4.05	2.66	5.99
ReS_2_	0HC	2H	3.31	2.40	2.88	PdS_2_	0HC	1T	3.55	2.40	2.48
	1HC	1T	3.32	2.42	4.35		1HC	1T	3.72	2.46	3.75
	2HC	1T	3.68	2.42	5.07		2HC	1T	3.94	2.53	4.96
ReSe_2_	0HC	2H	3.46	2.52	3.08	PdSe_2_	0HC	1T	3.74	2.52	2.61
	1HC	1T	3.86	2.54	3.93		1HC	1T	3.91	2.58	4.01
	2HC	1T	3.89	2.54	5.43		2HC	1T	4.10	2.64	5.36
ReTe_2_	0HC	2H	3.71	2.70	3.30	PdTe_2_	0HC	1T	4.03	2.70	2.75
	1HC	1T	4.09	2.71	4.37		1HC	1T	4.14	2.75	6.44
	2HC	1T	4.15	2.70	5.97		2HC	1T	4.31	2.80	6.01
						PtS_2_	0HC	1T	3.58	2.40	2.46
							1HC	1T	3.93	2.58	3.99
							2HC	1T	4.14	2.64	5.29
						PtSe_2_	0HC	1T	3.75	2.53	2.62
							1HC	1T	3.93	2.58	3.99
							2HC	1T	4.13	2.64	5.30
						PtTe_2_	0HC	1T	4.02	2.71	2.78
							1HC	1T	4.15	2.74	4.44
							2HC	1T	4.30	2.79	6.02

**Table 2:** Electronic, and magnetic ground states and magnetic moments of MX_2_-nHC (n = 0, 1, and 2).

Group IV	Structure	Conductivity	Magnetic state	Magnetic moment of M atom (μ_B_)	Magnetic moments of X atom & X atom with H (μ_B_)	Curie Temperature (K)	Exchange Energy (meV)
TiS_2_	1T-0HC	semiconductor	NM	—	—	—	0
	2H-1HC	semiconductor	FM	0.46	0.006 & 0.007	149	19
	2H-2HC	semiconductor	NM	—	—	—	0
TiSe_2_	1T-0HC	metal	NM	—	—	—	0
	2H-1HC	semiconductor	FM	0.62	0.007 & 0.020	297	38
	2H-2HC	semiconductor	NM	—	—	—	0
TiTe_2_	1T-0HC	metal	NM	—	—	—	0
	2H-1HC	semiconductor	FM	0.71	0.015 & 0.030	448	58
	2H-2HC	semiconductor	NM	—	—	—	0
ZrS_2_	1T-0HC	semiconductor	NM	—	—	—	0
	2H-1HC	half-metal	FM	0.40	0.027 & 0.003	90	12
	2H-2HC	semiconductor	NM	—	—	—	0
ZrSe_2_	1T-0HC	semiconductor	NM	—	—	—	0
	2H-1HC	half-metal	FM	0.42	0.013 & 0.002	91	12
	2H-2HC	semiconductor	NM	—	—	—	0
ZrTe_2_	1T-0HC	metal	NM	—	—	—	0
	2H-1HC	semiconductor	NM	—	—	—	0
	2H-2HC	semiconductor	NM	—	—	—	0
HfS_2_	1T-0HC	semiconductor	NM	—	—	—	0
	2H-1HC	metal	NM	—	—	—	0
	2H-2HC	semiconductor	NM	—	—	—	0
HfSe_2_	1T-0HC	semiconductor	NM	—	—	—	0
	2H-1HC	semiconductor	NM	—	—	—	0
	2H-2HC	metal	NM	—	—	—	0
HfTe_2_	1T-0HC	metal	NM	—	—	—	0
	2H-1HC	metal	NM	—	—	—	2
	2H-2HC	metal	NM	—	—	—	0

Non-magnetism, ferromagnetism, and anti-ferromagnetism are denoted as NM, FM and AFM, respectively.

**Table 3:** Electronic, and magnetic ground states and magnetic moments of MX_2_-nHC (n = 0, 1, and 2).

Group VI	Structure	Conductivity	Magnetic state	Magnetic moment of M atom (μ_B_)	Magnetic moments of X atom & X atom with H (μ_B_)	Curie Temperature (K)	Exchange Energy (meV)
CrS_2_	2H-0HC	semiconductor	NM	—	—	—	0
	2H-1HC	metal	AFM	2.27	0.044 & 0.021	—	−202
	1T-2HC	half-metal	FM	1.97	0.018	1317	170
CrSe_2_	2H-0HC	semiconductor	NM	—	—	—	0
	2H-1HC	metal	AFM	2.51	0.051 & 0.006	—	−137
	1T-2HC	metal	FM	2.11	0.039	1012	131
CrTe_2_	2H-0HC	semiconductor	AFM	1.42	0.056	—	−50
	2H-1HC	metal	AFM	2.67	0.065 & 0.027	—	−90
	1T-2HC	metal	FM	2.62	0.089	596	77
MoS_2_	2H-0HC	semiconductor	NM	—	—	—	0
	2H-1HC	semiconductor	NM	—	—	—	2
	1T-2HC	metal	NM	—	—	—	0
MoSe_2_	2H-0HC	semiconductor	NM	—	—	—	0
	2H-1HC	semiconductor	NM	—	—	—	0
	1T-2HC	metal	PM	1.05	0.008	—	−6
MoTe_2_	2H-0HC	semiconductor	NM	—	—	—	0
	2H-1HC	metal	NM	—	—	—	6
	1T-2HC	half-metal	FM	1.61	0.042	1096	12
WS_2_	2H-0HC	semiconductor	NM	—	—	—	0
	2H-1HC	semiconductor	NM	—	—	—	0
	1T-2HC	metal	NM	—	—	—	0
WSe_2_	2H-0HC	semiconductor	NM	—	—	—	0
	2H-1HC	semiconductor	NM	—	—	—	0
	1T-2HC	metal	NM	—	—	—	0
WTe_2_	2H-0HC	semiconductor	NM	—	—	—	0
	2H-1HC	metal	NM	—	—	—	5
	1T-2HC	half-metal	FM	1.48	0.046	1137	147

Non-magnetism, paramagnetism, ferromagnetism, and anti-ferromagnetism are denoted as NM, PM, FM and AFM, respectively.

**Table 4:** Electronic, and magnetic ground states and magnetic moments of MX_2_-nHC (n = 0, 1, and 2).

Group VII	Structure	Conductivity	Magnetic state	Magnetic moment of M atom (μ_B_)	Magnetic moments of X atom & X atom with H (μ_B_)	Curie Temperature (K)	Exchange Energy (meV)
TcS_2_	2H-0HC	metal	NM	—	—	—	0
	1T-1HC	metal	NM	—	—	—	0
	1T-2HC	half-metal	FM	0.94	0.002	541	70
TcSe_2_	2H-0HC	metal	NM	—	—	—	1
	1T-1HC	half-metal	FM	1.66	0.052 & 0.045	613	80
	1T-2HC	half-metal	FM	0.88	0.012	117	15
TcTe_2_	2H-0HC	metal	NM	—	—	—	4
	1T-1HC	metal	NM	—	—	—	6
	1T-2HC	metal	PM	0.61	0.004	—	−2
ReS_2_	2H-0HC	metal	NM	—	—	—	2
	1T-1HC	metal	NM	—	—	—	0
	1T-2HC	half-metal	FM	0.89	0.001	454	59
ReSe_2_	2H-0HC	metal	NM	—	—	—	4
	1T-1HC	half-metal	FM	1.51	0.051 & 0.036	537	69
	1T-2HC	half-metal	FM	0.84	0.009	204	26
ReTe_2_	2H-0HC	metal	NM	—	—	—	1
	1T-1HC	metal	NM	—	—	—	1
	1T-2HC	metal	NM	—	—	—	−3

Non-magnetism, paramagnetism, ferromagnetism, and anti-ferromagnetism are denoted as NM, PM, FM and AFM, respectively.

**Table 5:** Electronic, and magnetic ground states and magnetic moments of MX_2_-nHC (n = 0, 1, and 2 & M is from group X).

Group X	Condition	Conductivity	Magnetic state	Magnetic moment of M atom (μ_B_)	Magnetic moments of X atom & X atom with H (μ_B_)	Curie Temperature (K)	Exchange Energy (meV)
NiS_2_	1T-0HC	semiconductor	NM	—	—	—	0
	1T-1HC	half-metal	FM	0.59	0.176 & 0.065	228	30
	1T-2HC	metal	AFM	1.04	0.067	—	−73
NiSe_2_	1T-0HC	semiconductor	NM	—	—	—	0
	1T-1HC	semiconductor	FM	0.41	0.108 & 0.026	233	30
	1T-2HC	metal	AFM	0.51	0.011	—	−14
NiTe_2_	1T-0HC	metal	NM	—	—	—	1
	1T-1HC	metal	NM	—	—	—	8
	1T-2HC	metal	NM	—	—	—	8
PdS_2_	1T-0HC	semiconductor	NM	—	—	—	0
	1T-1HC	semiconductor	FM	0.33	0.220 & 0.054	197	25
	1T-2HC	metal	NM	—	—	—	0
PdSe_2_	1T-0HC	semiconductor	NM	—	—	—	0
	1T-1HC	semiconductor	FM	0.24	0.170 & 0.031	171	22
	1T-2HC	metal	NM	—	—	—	0
PdTe_2_	1T-0HC	semiconductor	NM	—	—	—	0
	1T-1HC	metal	NM	—	—	—	2
	1T-2HC	metal	NM	—	—	—	0
PtS_2_	1T-0HC	semiconductor	NM	—	—	—	0
	1T-1HC	semiconductor	FM	0.29	0.146 & 0.040	195	25
	1T-2HC	metal	NM	—	—	—	0
PtSe_2_	1T-0HC	semiconductor	NM	—	—	—	0
	1T-1HC	semiconductor	FM	0.29	0.146 & 0.040	195	25
	1T-2HC	metal	NM	—	—	—	0
PtTe_2_	1T-0HC	semiconductor	NM	—	—	—	0
	1T-1HC	metal	NM	—	—	—	1
	1T-2HC	metal	NM	—	—	—	0

Non-magnetism, ferromagnetism, and anti-ferromagnetism are denoted as NM, FM and AFM, respectively.
