# Supplementary material for: Hydrogenation-controlled phase transition on two-dimensional transition metal dichalcogenides and their unique physical and catalytic properties
Source: Sci Rep. 2016 Sep 30;6:34186. doi: 10.1038/srep34186 (PMC5043243; doi:10.1038/srep34186)
Supplement: Supplementary Information [file srep34186-s1.doc]

Hydrogenation-controlled phase transition on two-dimensional transition metal dichalcogenides and their unique physical and catalytic properties

Yuanju Qu^1,2^, Hui Pan^1^*, and Chi Tat Kwok^2,1^

^1^Institute of Applied Physics and Materials Engineering, Faculty of Science and Technology, University of Macau, Macao SAR, P. R. China

^2^ Department of Electromechanical Engineering, Faculty of Science and Technology, University of Macau, Macao SAR, P. R. China

*H. Pan ([huipan@umac.mo](mailto:huipan@umac.mo)); Tel: (853)88224427; Fax: (853)88222426

**
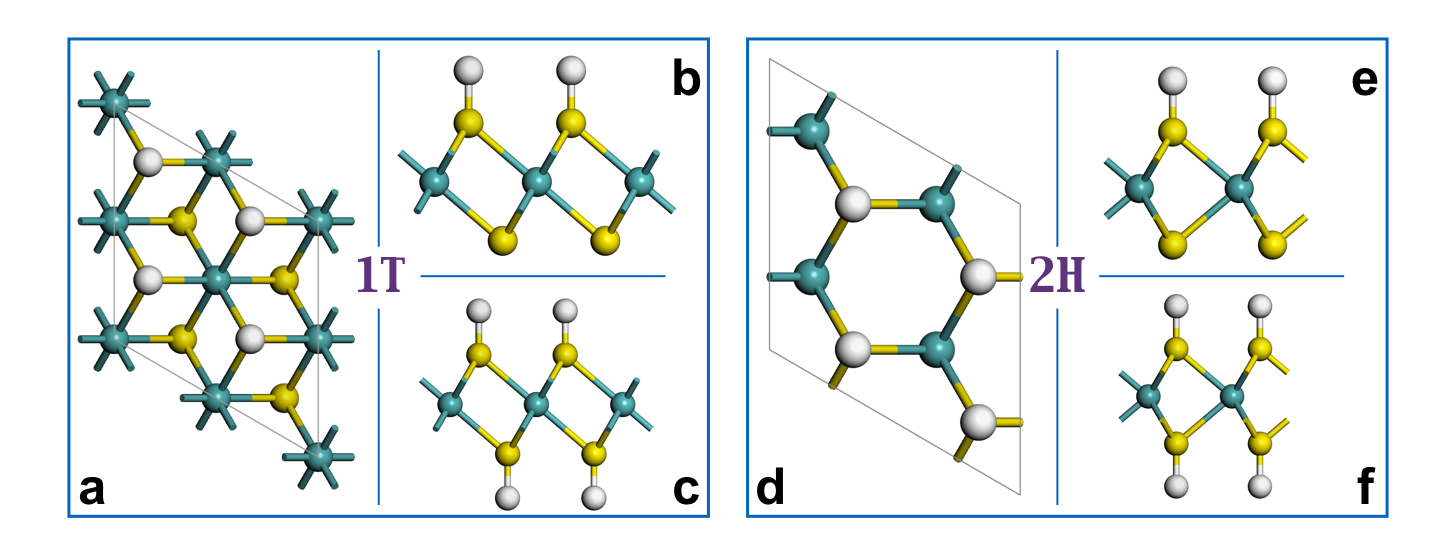
**

S1, The representative structures of MX_2_ monolayers in 221 supercells: (a) top view of 1T phase with one- or two-surface hydrogenation, (b) side view of 1T phase with one-surface hydrogenation, (c) side view of 1T phase with two-surface hydrogenation, (d) top view of 2H-phase with one- or two-surface hydrogenation, (e) side view of 2H phase with one-surface hydrogenation, (f) side view of 2H phase with two-surface hydrogenation.


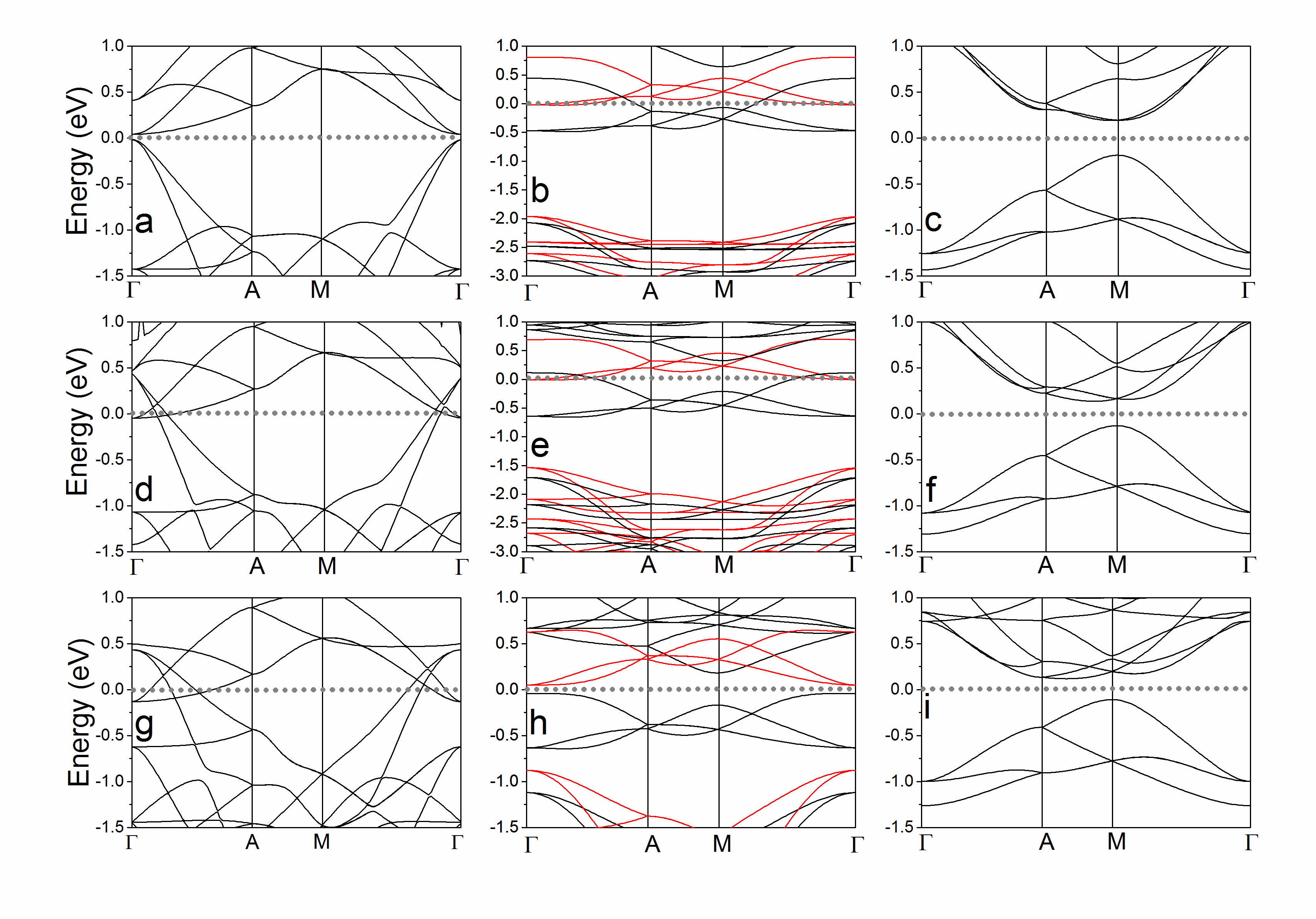
S2, Calculated band structures of (a) TiS_2_-0HC, (b) TiS_2_-1HC, (c) TiS_2_- 2HC, (d) TiSe_2_-0HC, (e) TiSe_2_-1HC, (f) TiSe_2_-2HC, (g) TiTe_2_-0HC, (h) TiTe_2_-1HC and (i) TiTe_2_-2HC monolayers. The Fermi level is at 0 eV and indicated by the dot line. The red and black lines in panel b, e & h represent spin-down and spin-up bands, respectively.


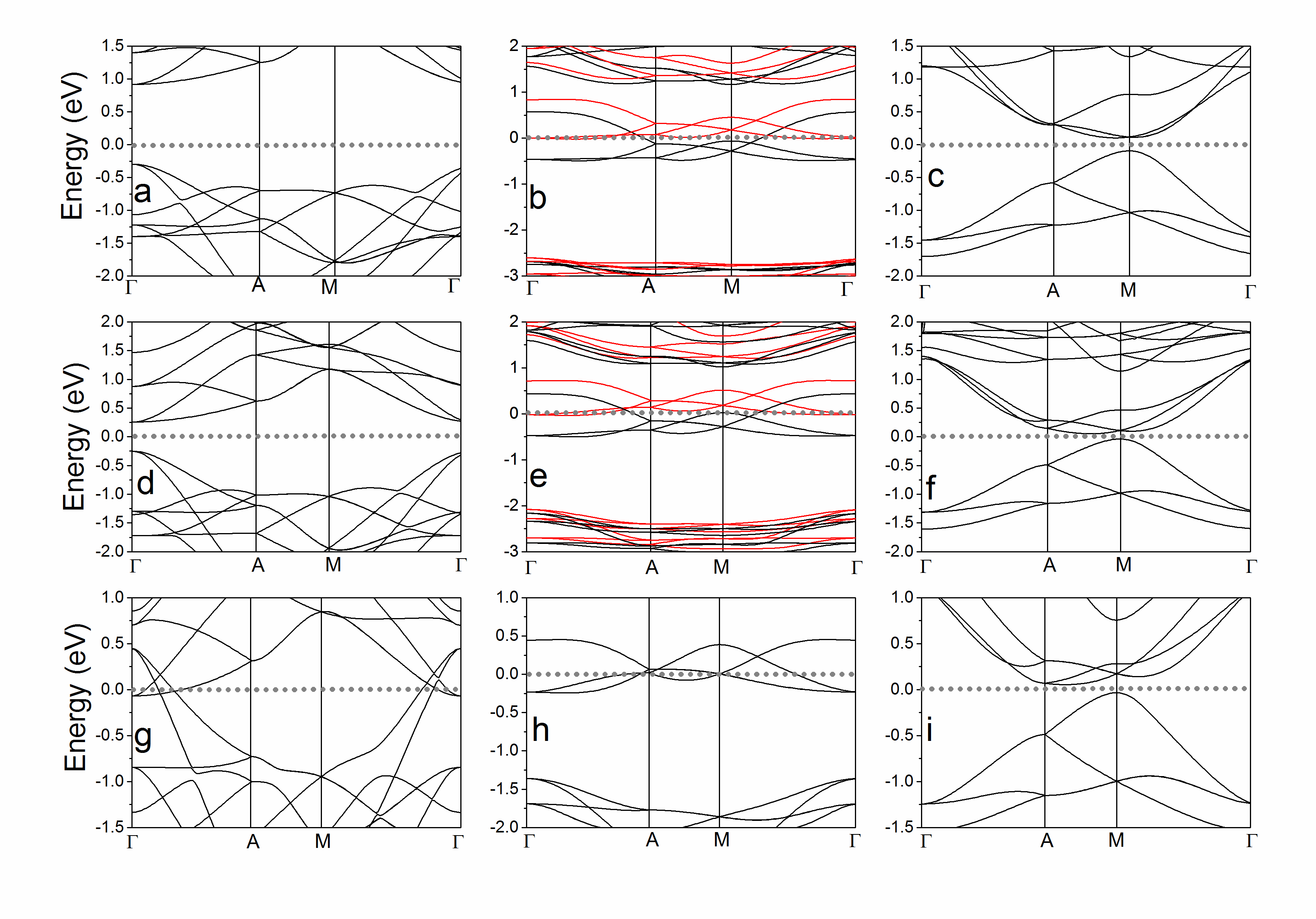


S3, Calculated band structures of (a) ZrS_2_-0HC, (b) ZrS_2_-1HC, (c) ZrS_2_- 2HC, (d) ZrSe_2_-0HC, (e) ZrSe_2_-1HC, (f) ZrSe_2_-2HC, (g) ZrTe_2_-0HC, (h) ZrTe_2_-1HC and (i) ZrTe_2_-2HC monolayers. The Fermi level is at 0 eV and indicated by the dot line. The red and black lines in panel b & e represent spin-down and spin-up bands, respectively.


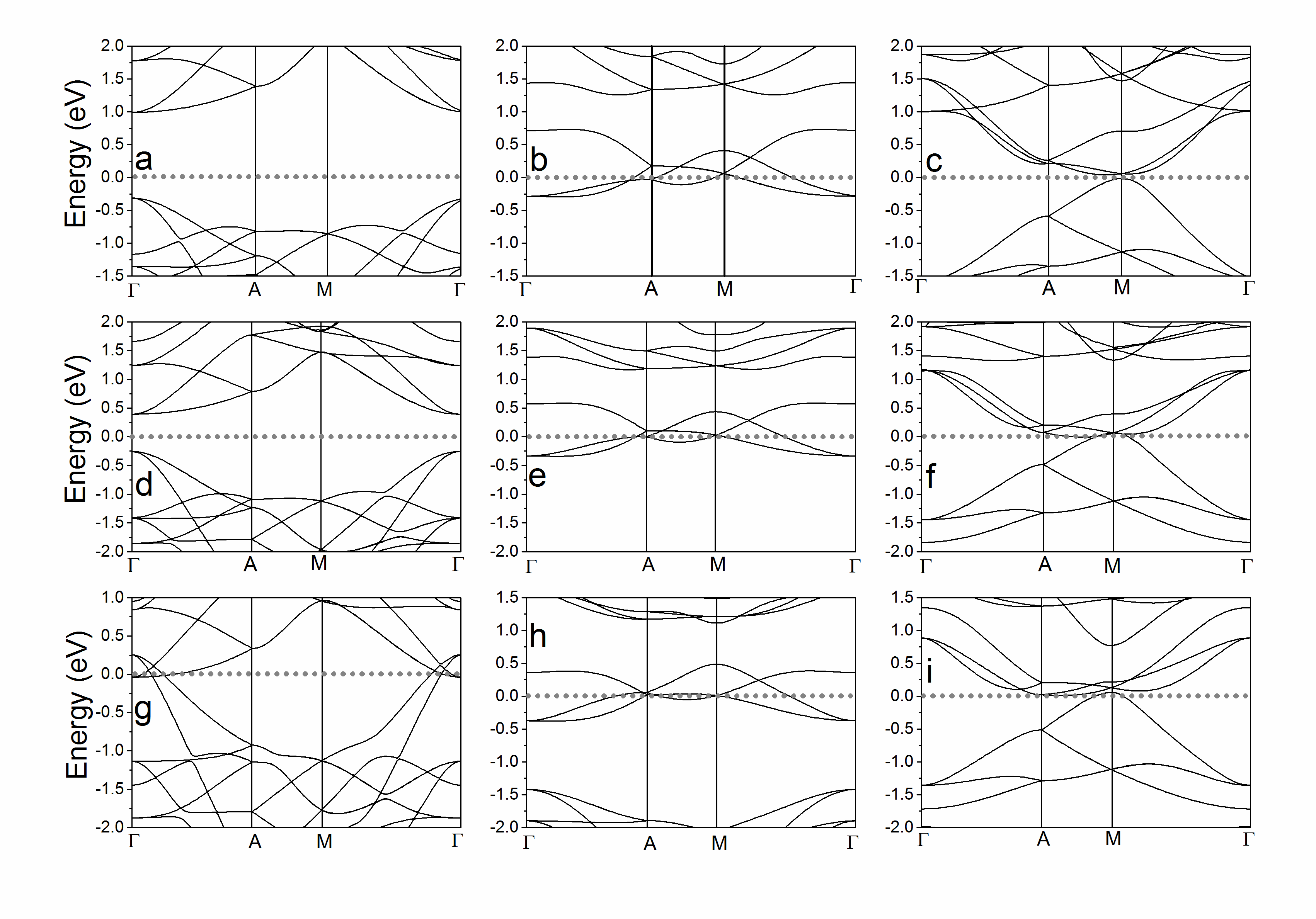


S4, Calculated band structures of (a) HfS_2_-0HC, (b) HfS_2_-1HC, (c) HfS_2_- 2HC, (d) HfSe_2_-0HC, (e) HfSe_2_-1HC, (f) HfSe_2_-2HC, (g) HfTe_2_-0HC, (h) HfTe_2_-1HC and (i) HfTe_2_-2HC monolayers. The Fermi level is at 0 eV and indicated by the dot line.


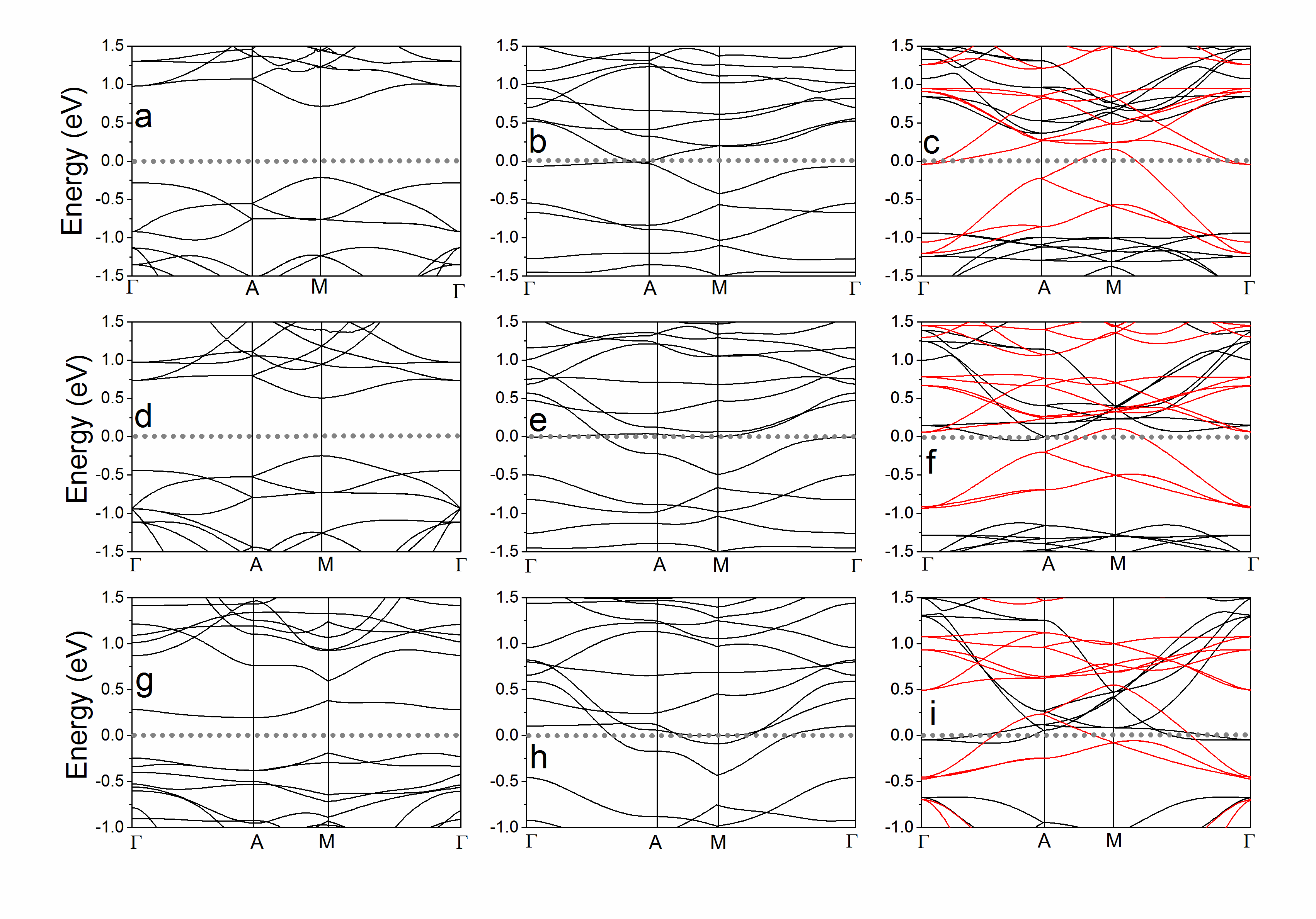


S5, Calculated band structures of (a) CrS_2_-0HC, (b) CrS_2_-1HC, (c) CrS_2_- 2HC, (d) CrSe_2_-0HC, (e) CrSe_2_-1HC, (f) CrSe_2_-2HC, (g) CrTe_2_-0HC, (h) CrTe_2_-1HC and (i) CrTe_2_-2HC monolayers. The Fermi level is at 0 eV and indicated by the dot line. The red and black lines in panel b, c e–i represent spin-down and spin-up bands, respectively.


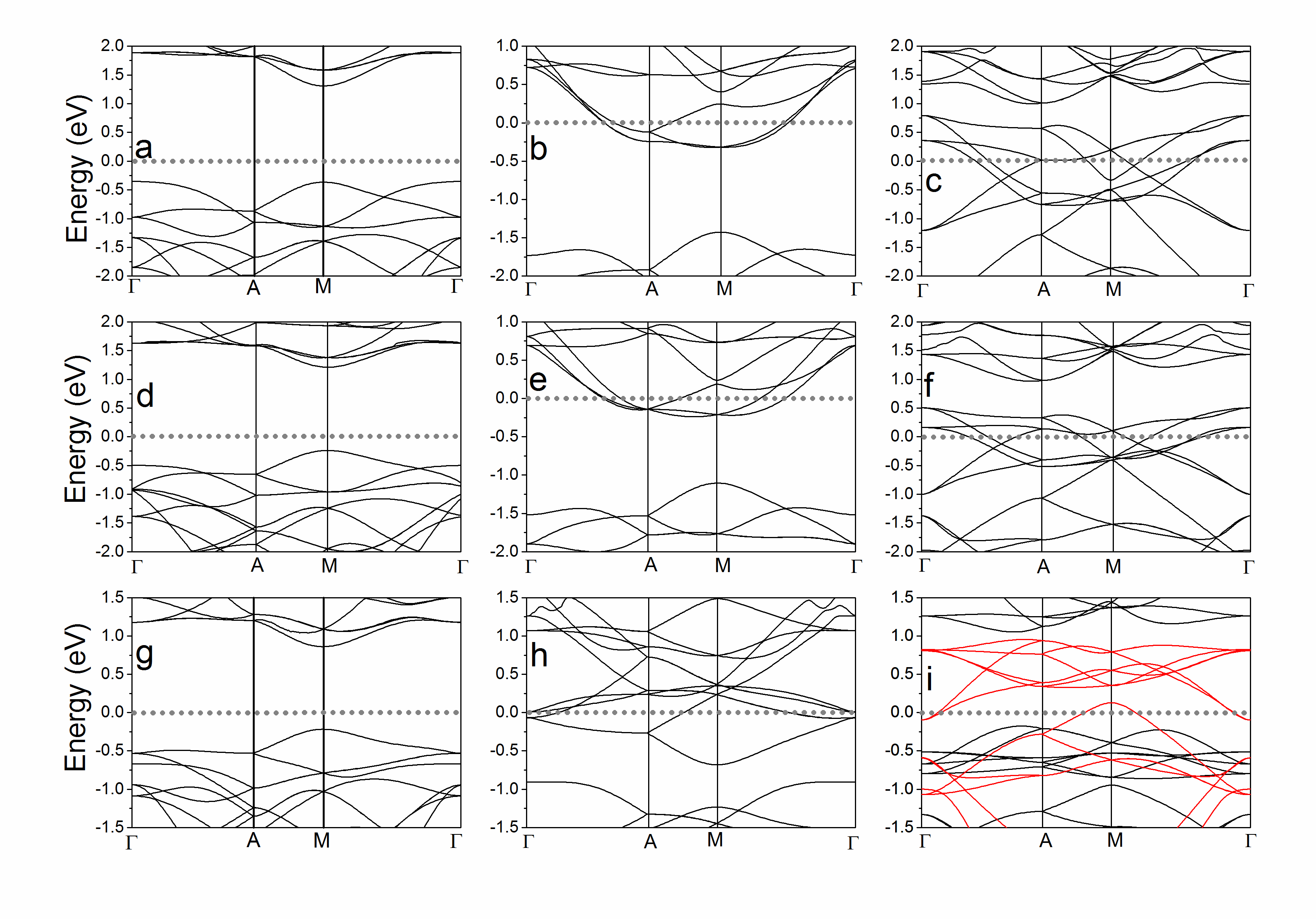
S6, Calculated band structures of (a) MoS_2_-0HC, (b) MoS_2_-1HC, (c) MoS_2_- 2HC, (d) MoSe_2_-0HC, (e) MoSe_2_-1HC, (f) MoSe_2_-2HC, (g) MoTe_2_-0HC, (h) MoTe_2_-1HC and (i) MoTe_2_-2HC monolayers. The Fermi level is at 0 eV indicated by the gray dot line. The red and black lines in panel i represent spin-down and spin-up bands, respectively.


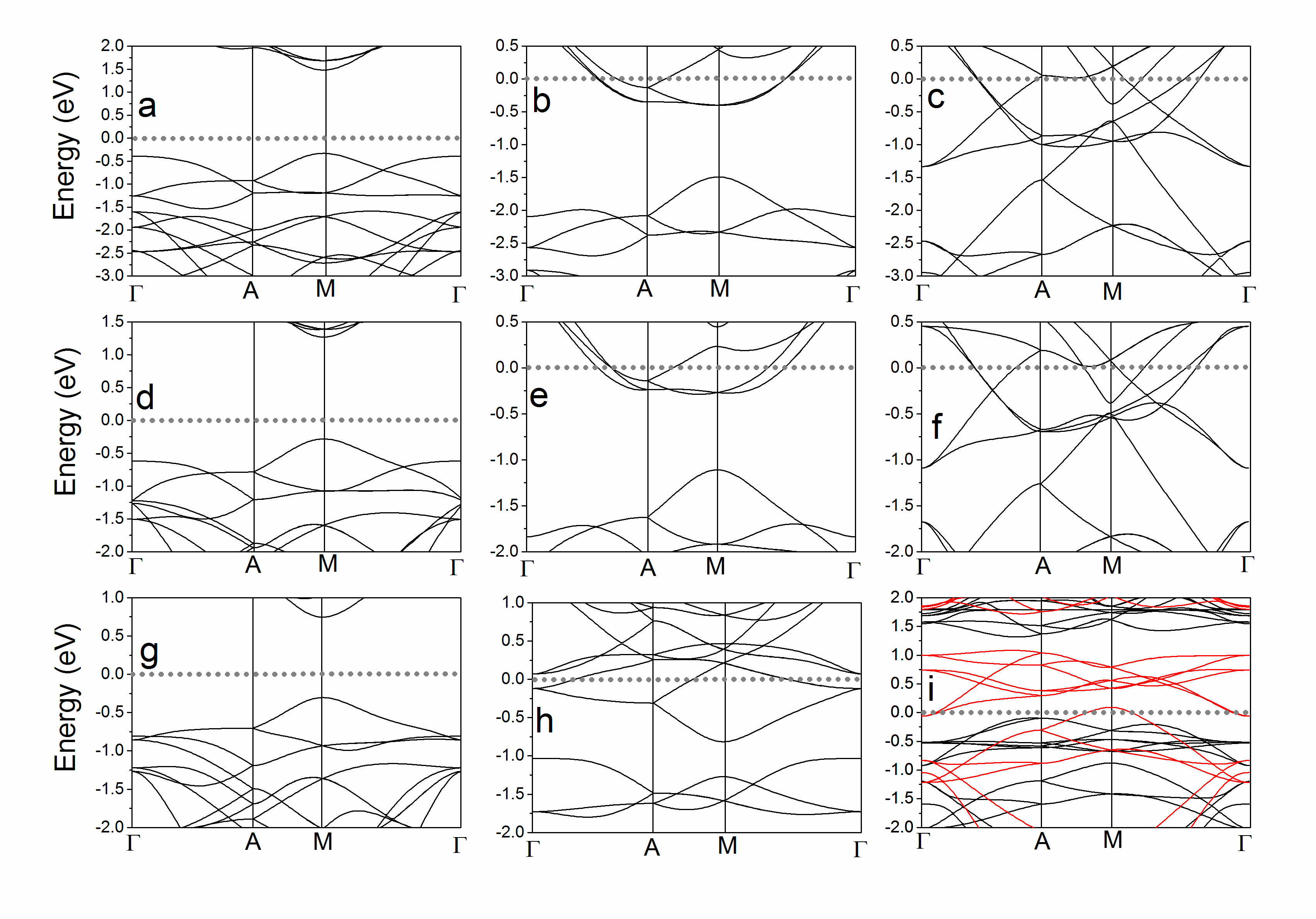


S7, Calculated band structures of (a) WS_2_-0HC, (b) WS_2_-1HC, (c) WS_2_- 2HC, (d) WSe_2_-0HC, (e) WSe_2_-1HC, (f) WSe_2_-2HC, (g) WTe_2_-0HC, (h) WTe_2_-1HC and (i) WTe_2_-2HC monolayers. The Fermi level is at 0 eV and indicated by the dot line. The red and black lines in panel i represent spin-down and spin-up bands, respectively.


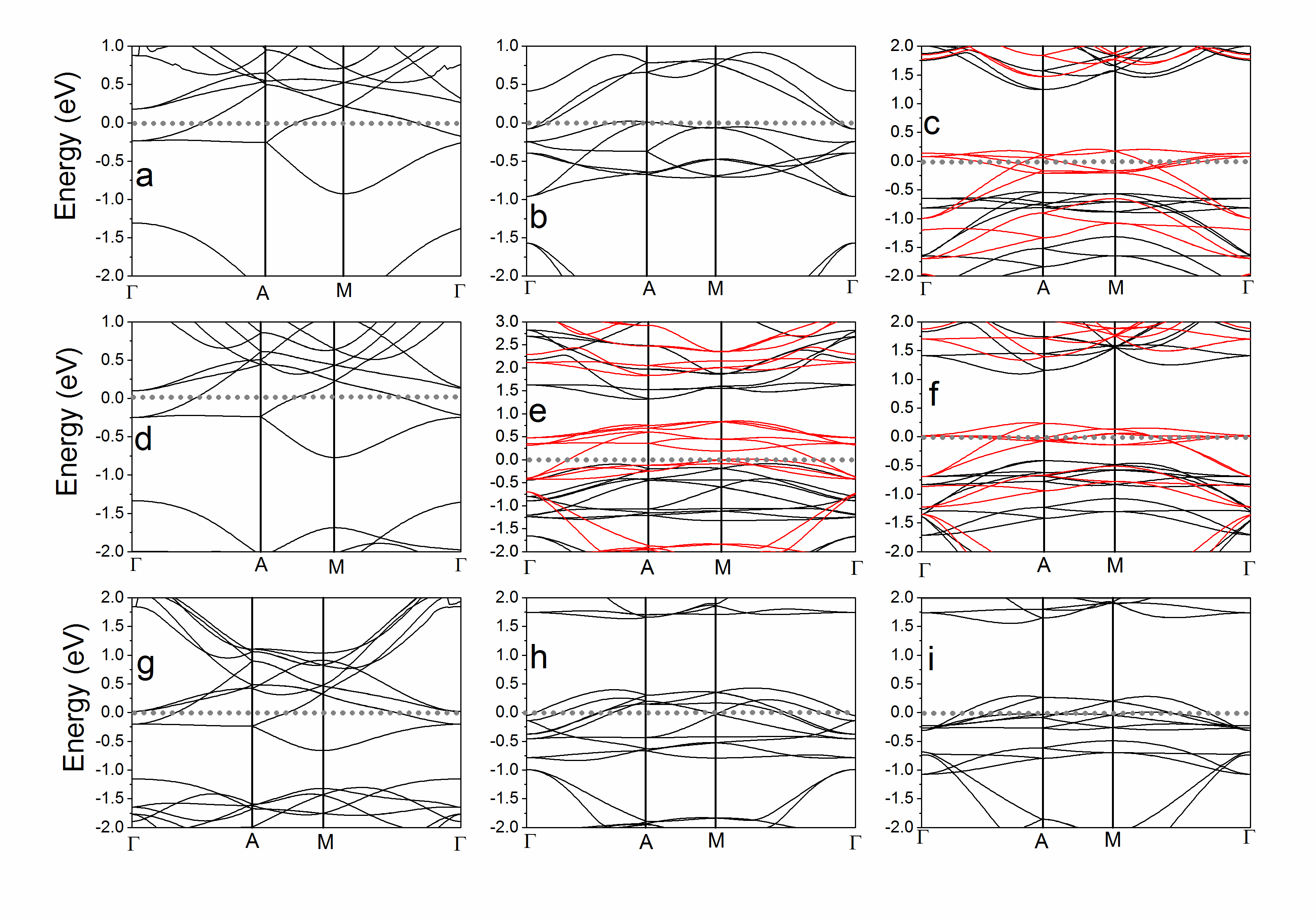


S8, Calculated band structures of (a) TcS_2_-0HC, (b) TcS_2_-1HC, (c) TcS_2_- 2HC, (d) TcSe_2_-0HC, (e) TcSe_2_-1HC, (f) TcSe_2_-2HC, (g) TcTe_2_-0HC, (h) TcTe_2_-1HC and (i) TcTe_2_-2HC monolayers. The Fermi level is at 0 eV and indicated by the dot line. The red and black lines in panel c, e & f represent spin-down and spin-up bands, respectively.


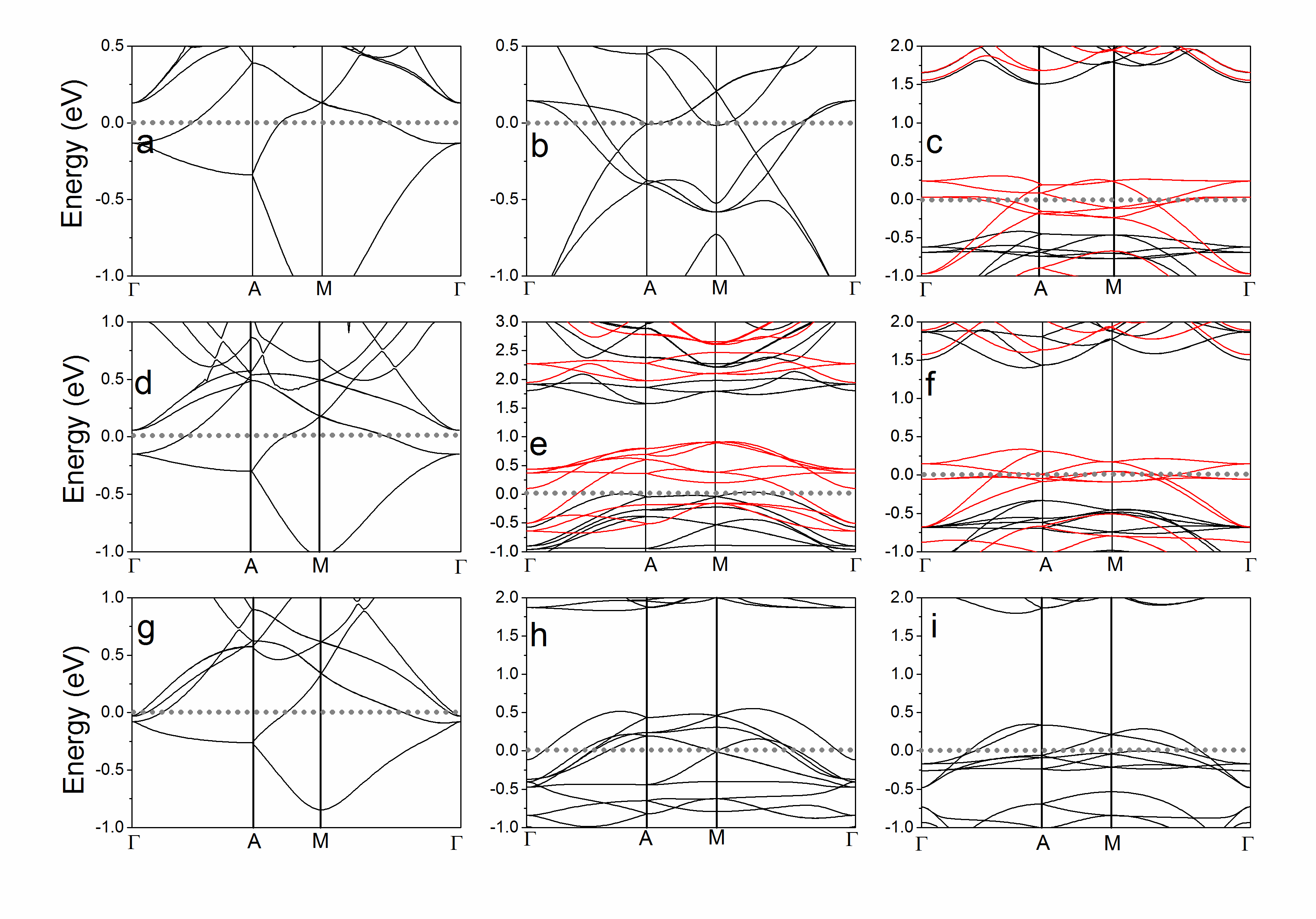


S9, Calculated band structures of (a) ReS_2_-0HC, (b) ReS_2_-1HC, (c) ReS_2_- 2HC, (d) ReSe_2_-0HC, (e) ReSe_2_-1HC, (f) ReSe_2_-2HC, (g) ReTe_2_-0HC, (h) ReTe_2_-1HC and (i) ReTe_2_-2HC monolayers. The Fermi level is at 0 eV and indicated by the dot line. The red and black lines in panel c, e & f represent spin-down and spin-up bands, respectively.


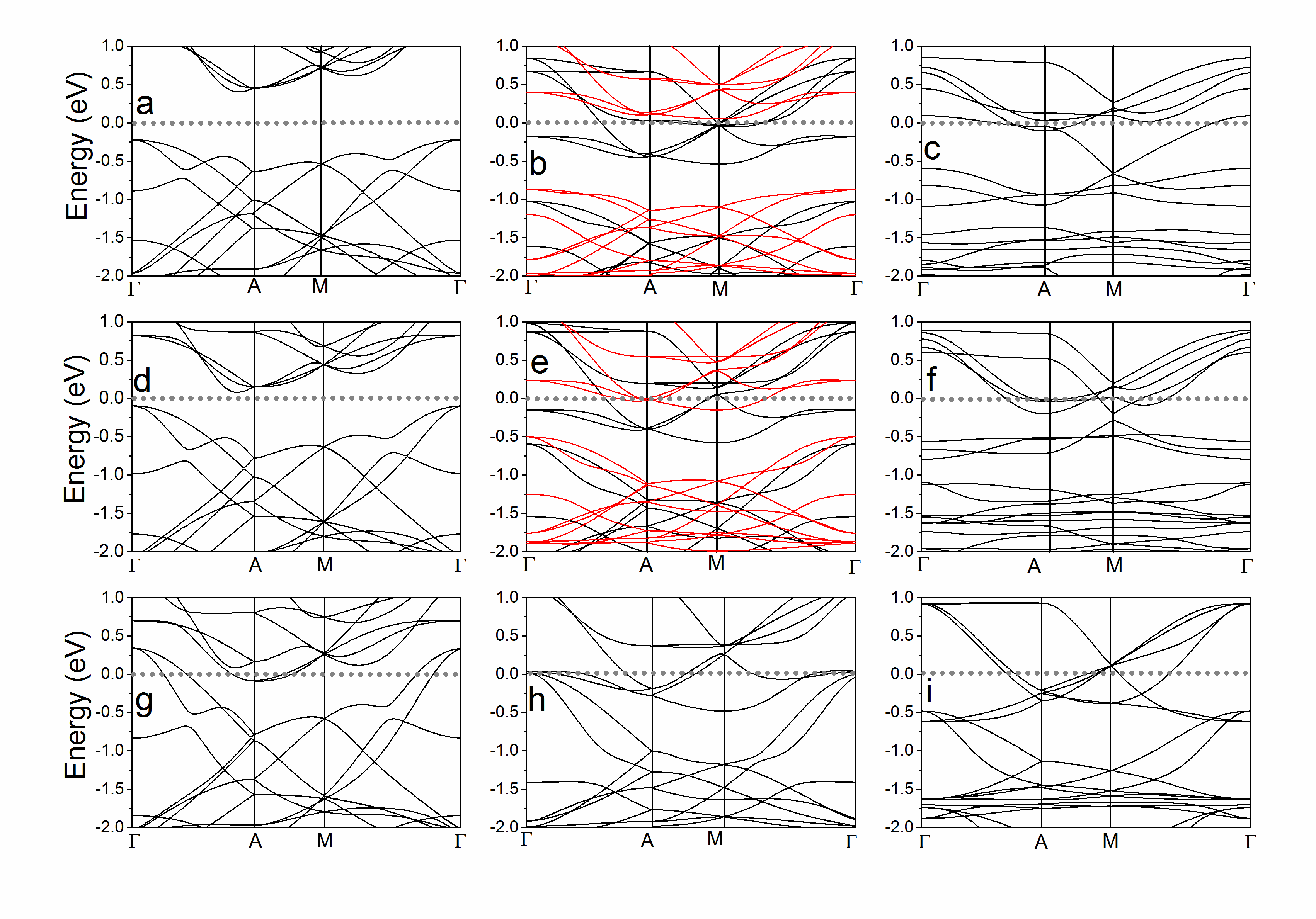
S10, Calculated band structures of (a) NiS_2_-0HC, (b) NiS_2_-1HC, (c) NiS_2_- 2HC, (d) NiSe_2_-0HC, (e) NiSe_2_-1HC, (f) NiSe_2_-2HC, (g) NiTe_2_-0HC, (h) NiTe_2_-1HC and (i) NiTe_2_-2HC monolayers. The Fermi level is at 0 eV and indicated by the dot line. The red and black lines in panels b, c, e & f represent spin-down and spin-up bands, respectively.


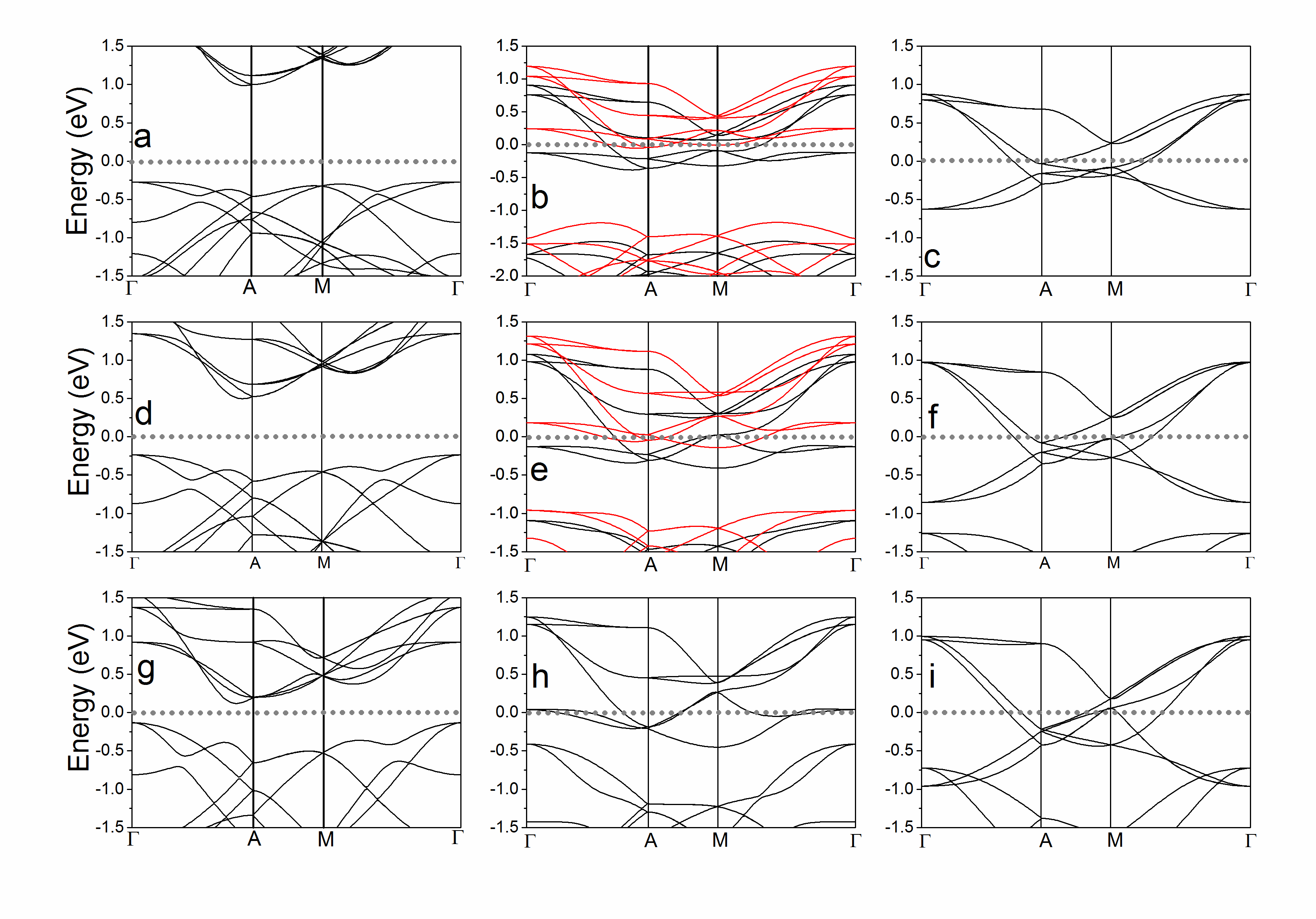
S11, Calculated band structures of (a) PdS_2_-0HC, (b) PdS_2_-1HC, (c) PdS_2_- 2HC, (d) PdSe_2_-0HC, (e) PdSe_2_-1HC, (f) PdSe_2_-2HC, (g) PdTe_2_-0HC, (h) PdTe_2_-1HC and (i) PdTe_2_-2HC monolayers. The Fermi level is at 0 eV and indicated by the dot line. The red and black lines in panel b & e represent spin-down and spin-up bands, respectively.


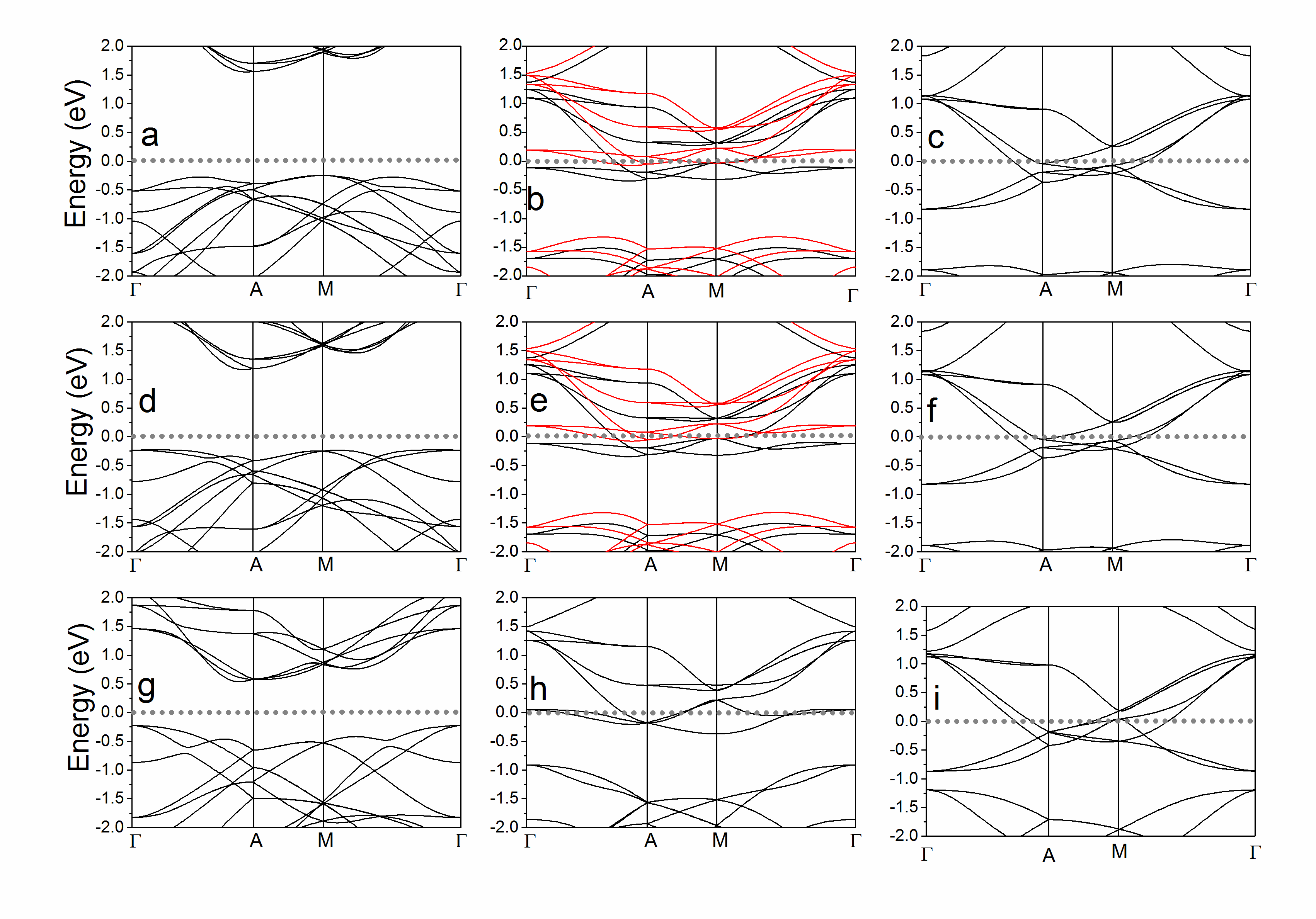
S12, Calculated band structures of (a) PtS_2_-0HC, (b) PtS_2_-1HC, (c) PtS_2_- 2HC, (d) PtSe_2_-0HC, (e) PtSe_2_-1HC, (f) PtSe_2_-2HC, (g) PtTe_2_-0HC, (h) PtTe_2_-1HC and (i) PtTe_2_-2HC monolayers. The Fermi level is at 0 eV and indicated by the dot line. The red and black lines in panel b & e represent spin-down and spin-up bands, respectively.


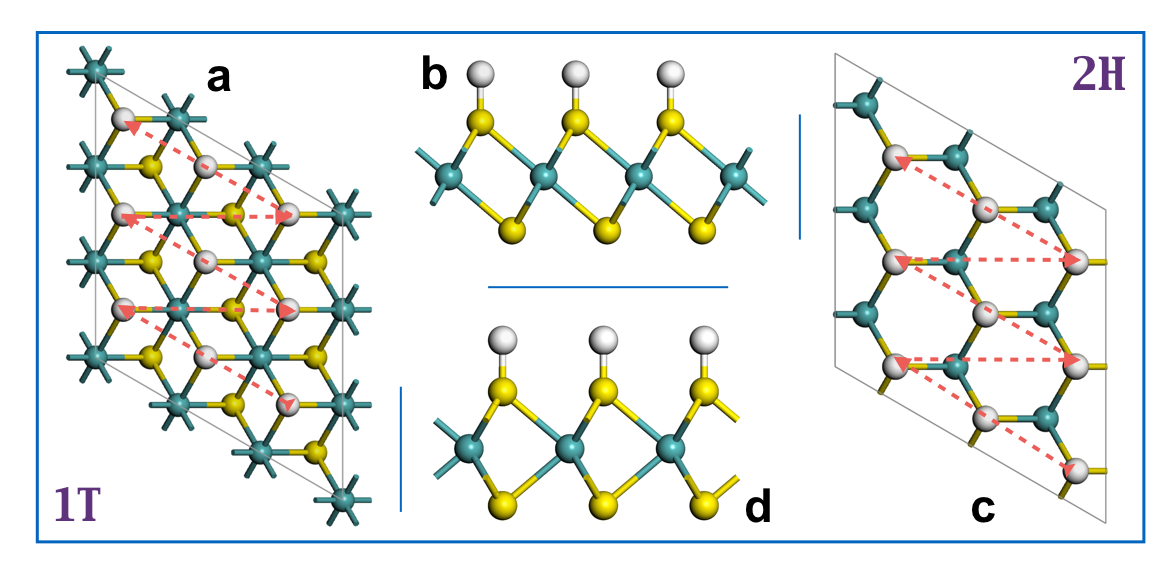


S13, The representative structures of MX_2_ monolayers in 331 supercells: (a) top and (b) side views of 1T phase with one-surface hydrogenation, (c) top and (d) side view of 2H phase with one-surface full hydrogenation. The red-dot line indicates the direction of the increment of hydrogen coverage.

Table S1 Calculated total energy differences between AFM and NM, FM and NM, and AFM and NM for the system with negligible exchange energies.

| Group  IV | Structure | E_AFM_ - E_NM_  (meV/unit cell) | E_FM_ - E_NM_  (meV/unit cell) | E_AFM_ - E_FM_  (meV/unit cell) |
| --- | --- | --- | --- | --- |
| TiS_2_ | 1T-0HC | 0 | 0 | 0 |
|  | 2H-2HC | 0 | 0 | 0 |
| TiSe_2_ | 1T-0HC | 0 | 0 | 0 |
|  | 2H-2HC | 0 | 0 | 0 |
| TiTe_2_ | 1T-0HC | 0 | 0 | 0 |
|  | 2H-2HC | 0 | 0 | 0 |
| ZrS_2_ | 1T-0HC | 0 | 0 | 0 |
|  | 2H-2HC | 0 | 0 | 0 |
| ZrSe_2_ | 1T-0HC | 0 | 0 | 0 |
|  | 2H-2HC | 0 | 0 | 0 |
| ZrTe_2_ | 1T-0HC | 0 | 0 | 0 |
|  | 2H-1HC | 0 | 0 | 0 |
|  | 2H-2HC | 0 | 0 | 0 |
| HfS_2_ | 1T-0HC | 0 | 0 | 1 |
|  | 2H-1HC | -3 | -3 | 0 |
|  | 2H-2HC | 0 | 0 | 0 |
| HfSe_2_ | 1T-0HC | 0 | 0 | 0 |
|  | 2H-1HC | -1 | -1 | 0 |
|  | 2H-2HC | 0 | 0 | 0 |
| HfTe_2_ | 1T-0HC | 0 | 0 | 0 |
|  | 2H-1HC | 0 | -2 | 2 |
|  | 2H-2HC | 0 | 0 | 0 |
| Group  V | Structure | E_AFM_ - E_NM_  (meV/unit cell) | E_FM_ - E_NM_  (meV/unit cell) | E_AFM_ - E_FM_  (meV/unit cell) |
| CrS_2_ | 2H-0HC | 0 | 0 | 0 |
| CrSe_2_ | 2H-0HC | 0 | 0 | 0 |
| MoS_2_ | 2H-0HC | 0 | 0 | 0 |
|  | 2H-1HC | 0 | -2 | 2 |
|  | 1T-2HC | 0 | 0 | 0 |
| MoSe_2_ | 2H-0HC | 0 | 0 | 0 |
|  | 2H-1HC | 0 | 0 | 0 |
|  | 1T-2HC | -33 | -27 | -6 |
| MoTe_2_ | 2H-0HC | 0 | 0 | 0 |
|  | 2H-1HC | 0 | -6 | 6 |
| WS_2_ | 2H-0HC | 0 | 0 | 0 |
|  | 2H-1HC | 0 | 0 | 0 |
|  | 1T-2HC | 0 | 0 | 0 |
| WSe_2_ | 2H-0HC | 0 | 0 | 0 |
|  | 2H-1HC | 0 | 0 | 0 |
|  | 1T-2HC | 0 | 0 | 0 |
| WTe_2_ | 2H-0HC | 0 | 0 | 0 |
|  | 2H-1HC | 0 | -6 | 5 |
| Group  VII | Structure | E_AFM_ - E_NM_  (meV/unit cell) | E_FM_ - E_NM_  (meV/unit cell) | E_AFM_ - E_FM_  (meV/unit cell) |
| TcS_2_ | 2H-0HC | 0 | 0 | 0 |
|  | 1T-1HC | 0 | 0 | 0 |
| TcSe_2_ | 2H-0HC | 0 | -1 | 1 |
| TcTe_2_ | 2H-0HC | 0 | -4 | 4 |
|  | 1T-1HC | 0 | -6 | 6 |
|  | 1T-2HC | -21 | -19 | -2 |
| ReS_2_ | 2H-0HC | 0 | -2 | 2 |
|  | 1T-1HC | 0 | 0 | 0 |
| ReSe_2_ | 2H-0HC | 0 | -4 | 4 |
| ReTe_2_ | 2H-0HC | 0 | -1 | 1 |
|  | 1T-1HC | 0 | -1 | 1 |
|  | 1T-2HC | -5 | -1 | -3 |
| Group  X | Structure | E_AFM_ - E_NM_  (meV/unit cell) | E_FM_ - E_NM_  (meV/unit cell) | E_AFM_ - E_FM_  (meV/unit cell) |
| NiS_2_ | 1T-0HC | 0 | 0 | 0 |
| NiSe_2_ | 1T-0HC | 0 | 0 | 0 |
| NiTe_2_ | 1T-0HC | 0 | -1 | 1 |
|  | 1T-1HC | 0 | -8 | 8 |
|  | 1T-2HC | 0 | -8 | 9 |
| PdS_2_ | 1T-0HC | 0 | 0 | 0 |
|  | 1T-2HC | 1 | 0 | 0 |
| PdSe_2_ | 1T-0HC | 0 | 0 | 0 |
|  | 1T-2HC | 0 | 0 | 0 |
| PdTe_2_ | 1T-0HC | 0 | 0 | 0 |
|  | 1T-1HC | 0 | -2 | 2 |
|  | 1T-2HC | 0 | 0 | 0 |
| PtS_2_ | 1T-0HC | 0 | 0 | 0 |
|  | 1T-2HC | 0 | 0 | 0 |
| PtSe_2_ | 1T-0HC | 0 | 0 | 0 |
|  | 1T-2HC | 0 | 0 | 0 |
| PtTe_2_ | 1T-0HC | 0 | 0 | 0 |
|  | 1T-1HC | 0 | -1 | 1 |
|  | 1T-2HC | 0 | 0 | 0 |
